# Effects of antipsychotics on brain insulin action in females: Study protocol for a placebo-controlled, crossover neuroimaging study

**DOI:** 10.1371/journal.pone.0352262

**Published:** 2026-08-27

**Authors:** Nicolette Stogios, Margaret K. Hahn, Gary Remington, Ariel Graff-Guerrero, Meng-Chuan Lai, Sri Mahavir Agarwal

**Affiliations:** 1 Schizophrenia Division, Centre for Addiction and Mental Health, Toronto, Ontario, Canada; 2 Department of Psychiatry, Temerty Faculty of Medicine, University of Toronto, Toronto, Ontario, Canada; 3 Banting and Best Diabetes Centre, University of Toronto, Toronto, Ontario, Canada; 4 Multimodal Imaging Group, Research Imaging Centre, Centre for Addiction and Mental Health, Toronto, Ontario, Canada; 5 Margaret and Wallace McCain Centre for Child, Youth & Family Mental Health and Azrieli Adult Neurodevelopmental Centre, Campbell Family Mental Health Research Institute, Centre for Addiction and Mental Health, Toronto, Ontario, Canada; Oita University Faculty of Medicine, JAPAN

## Abstract

Antipsychotic medications can cause serious metabolic adverse effects, including type 2 diabetes, with females disproportionally more affected than males. Recently, brain insulin resistance (IR) has been posited as a potential determinant of these effects. Currently there are no direct methods to investigate brain IR, but physiological response to intranasal insulin (INI) can be used as a surrogate marker. INI reliably suppresses endogenous glucose production and modulates resting-state brain activity in humans; however, the current literature has been heavily focused on males. Recently, it has been reported that hormonal fluctuations throughout the menstrual cycle may influence brain insulin sensitivity. Brain insulin sensitivity is greatest in lean females during the follicular phase, matching that of lean males, but decreases during the luteal phase. This study aims to investigate brain insulin action in females across different phases of the menstrual cycle, and to determine how a metabolically high-risk antipsychotic medication, olanzapine, may disrupt these effects. In a single-blind, crossover design, fifteen female participants aged 18–35 years will undergo a neuroimaging-based assay of brain insulin action with oral olanzapine (or placebo) and intranasal insulin (or placebo) as pharmacological challenges. All treatment conditions will be administered once in each menstrual phase, meaning that each participant receives every condition twice over the course of the study. Two visits will take place during the follicular phase of the menstrual cycle (i.e., cycle days 3–11), and another two visits during the luteal phase (i.e., cycle days 18–25). During each study period, participants will receive either oral olanzapine (OLA) or oral placebo (PL) at doses of 5 mg on Day 0 and 10 mg on Day 1. On Day 2, participants will undergo two MRI scans, the first following intranasal placebo (INP) administration and the second following intranasal insulin (INI). It is hypothesized that INI, but not INP, will modulate resting state brain activity during the follicular menstrual cycle phase but not luteal phase. Oral OLA will subsequently inhibit all INI-induced effects, relative to oral PL, during the follicular phase; OLA will have no apparent effect in the luteal phase given pre-existing brain IR. This is the first study to explore the effect of menstrual cycle phases on the anti-insulin action of antipsychotics. This investigation is necessary for the development of suitable treatment options to minimize the metabolic impact of antipsychotics in females.

**Trial registration:**

ClinicalTrials.gov Registration: NCT06251635

## Introduction

Antipsychotic (AP) medications carry an adverse metabolic side effect profile which significantly increases the risk of developing type 2 diabetes and cardiovascular disease by 3–5-fold in patients using these medications compared to the general population.[1–6] These adverse effects accrue early during treatment; alarmingly high rates of dyslipidemia (>55%) and prediabetes (>15%) have been reported in first episode patients within 6 months of AP exposure.[7] Of particular concern is that females are disproportionally more affected by these adverse effects than males; [8,9] findings from the CATIE study revealed that the prevalence of metabolic syndrome in patients with schizophrenia spectrum disorder (SSD) was 51.6% among females compared to 36% among males.[10] AP-associated metabolic disturbances are linked to non-adherence, lower self-esteem, and decreased quality of life, [11] and might exacerbate pre-existing cognitive dysfunction in SSD.[12] These cumulative effects significantly increase the illness burden of SSD. Notably, there is evidence to suggest that females with SSD tend to report lower quality of life than males with SSD, despite having less severe course of the illness.[13] It is possible their increased susceptibility for the metabolic side effects of antipsychotics may contribute to the reported lower quality of life than males.

While the pathophysiology of these effects remains unclear, brain insulin resistance (IR) is posited to be an important determinant [14,15]. Insulin receptors are widely expressed in the brain and bear on multiple physiologic processes [14]. For instance, insulin, separate from its effects in the periphery, regulates hepatic glucose production through insulin receptors in the hypothalamus. Using the pancreatic euglycemic clamp technique to separate central and peripheral insulin effects, it has been established that an intracerebroventricular (ICV) infusion of insulin in male rodents results in a decrease in glucose production by the liver [16,17]. Similar findings have been observed in healthy male humans with intranasal insulin (INI) (which is a direct, non-invasive way of delivering insulin to the brain, analogous to ICV infusions in rodents) [18].

Our group has shown that an acute dose of olanzapine (OLA) can completely abolish this well-established ability of a central insulin stimulus to decrease hepatic glucose production in male rats [19]. This suggests that antipsychotics have a direct anti-insulin action *in the brain* that is independent of weight gain. Despite reported sex differences in the effects of brain insulin on energy balance in the literature where males are believed to be more sensitive to the catabolic actions of insulin than females [20,21], this finding is yet to be explored in females. Preclinical evidence has demonstrated that ICV infusion of insulin decreases food intake and body weight in male rats but not female rats, male rats given estradiol, or ovariectomized female rats [22]. This suggests that insulin action in the brain is sex-dependent and requires further sex-focused investigation.

Several neuroimaging studies have employed INI to demonstrate the effects of insulin on the brain. For example, in a study including both sexes, INI enhanced connectivity in the default mode network (DMN), measured using resting state functional magnetic resonance imaging (rs-fMRI) [23]. This ability of INI to induce changes in MRI-based parameters in healthy humans allows it to be leveraged as an assay of insulin action in the brain [24]. Lack of expected responses to an INI challenge is interpreted as a lack of insulin sensitivity. However, these results are primarily based on male-only studies, with a limited number of studies examining only females or both sexes.

To this last point, there continues to be a significant sex bias in our understanding of insulin effects in the brain as most of the research with INI has been conducted in males alone [18,25–30]. In the few studies that have included both males and females, differential effects according to sex have been noted. For example, INI appears to acutely reduce food intake in fasted, normal-weight males, but not females [31]; however, INI has been shown to increase satiety and reduce the palatability of certain foods in females [32]. Similarly, 8 weeks of INI treatment resulted in a significant reduction in body weight and body fat content in males only [21].

A recent report has indicated that brain insulin sensitivity differs between sexes, and this may relate to menstrual phases [33]. Specifically, brain insulin sensitivity in females is higher and comparable to lean males during the follicular phase (i.e., right before ovulation when estrogen levels are high) than during the luteal phase (i.e., after ovulation when both estrogen and progesterone levels are high) [33]. and this may contribute to the well-established decrease in whole-body insulin sensitivity in the luteal phase [34,35].

There has yet to be a study that investigates AP-induced insulin resistance in females and the impact of menstrual cycle factors on this relationship. Preclinical evidence of AP-induced disruption in brain insulin action was in male rats only, and previous work aimed at translating these findings did not investigate the effect of menstrual phase [24] It is therefore critical to study the effect of APs on brain insulin action in females, and to examine how it fluctuates with the menstrual cycle.

## Objectives and hypotheses

In this study, we seek to address a significant gap in the literature by investigating 1) how brain insulin action differs in females according to their menstrual cycle phase, and 2) how a high metabolic liability agent such as OLA might interrupt these differential insulin effects. We have chosen to conduct this study in healthy volunteers to minimize confounding effects of psychiatric disorders on glucose metabolism [36]. OLA was selected as a representative antipsychotic due to its well-established metabolic liability [37]. An acute dosing paradigm (i.e., two days of dosing) is employed to avoid longer-term changes in adiposity [38]. thereby allowing for a more precise assessment of whether antipsychotics exert direct effects on brain insulin function.

Resting state functional activity and connectivity (rsFC) will be used as a neural signature of brain insulin action. We hypothesize that 1) INI, but not intranasal placebo (INP), will modulate resting state activity in the brain in females during the follicular phase of their menstrual cycle but not the luteal phase; 2) Oral OLA will inhibit all INI-induced effects, relative to oral placebo (PL) during the follicular phase. OLA will have no apparent effect in the luteal phase given pre-existing brain IR.

## Methods and materials

**Fig 1:**
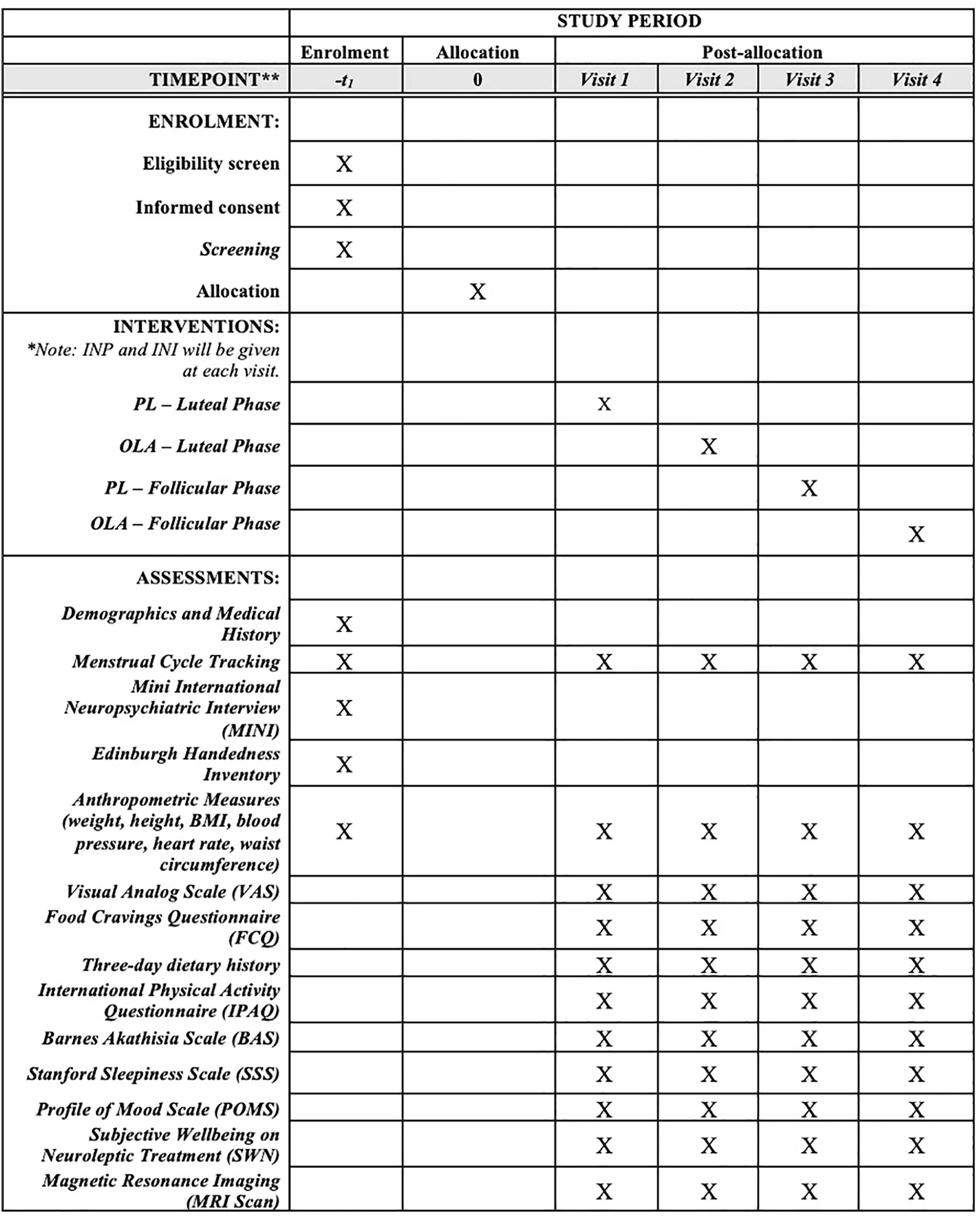
SPIRIT schedule of enrollment, interventions, and assessments.

The study is a single centre clinical trial conducted at the Centre for Addiction and Mental Health (CAMH), a psychiatric teaching hospital in Toronto, Canada. It has been granted full ethics approval by the CAMH Research Ethics Board (REB Protocol #127/2023, Version #5, 11-October-2024; S1 File). This study is also approved under the purview of Health Canada and has been registered with ClinicalTrials.gov (NCT06251635). The Standard Protocol Items: Recommendations for Interventional Trials (SPIRIT) schedule of enrolment is reported in Fig 1. The SPIRIT checklist is available in S2 File.

### Study design

**Fig 2:**
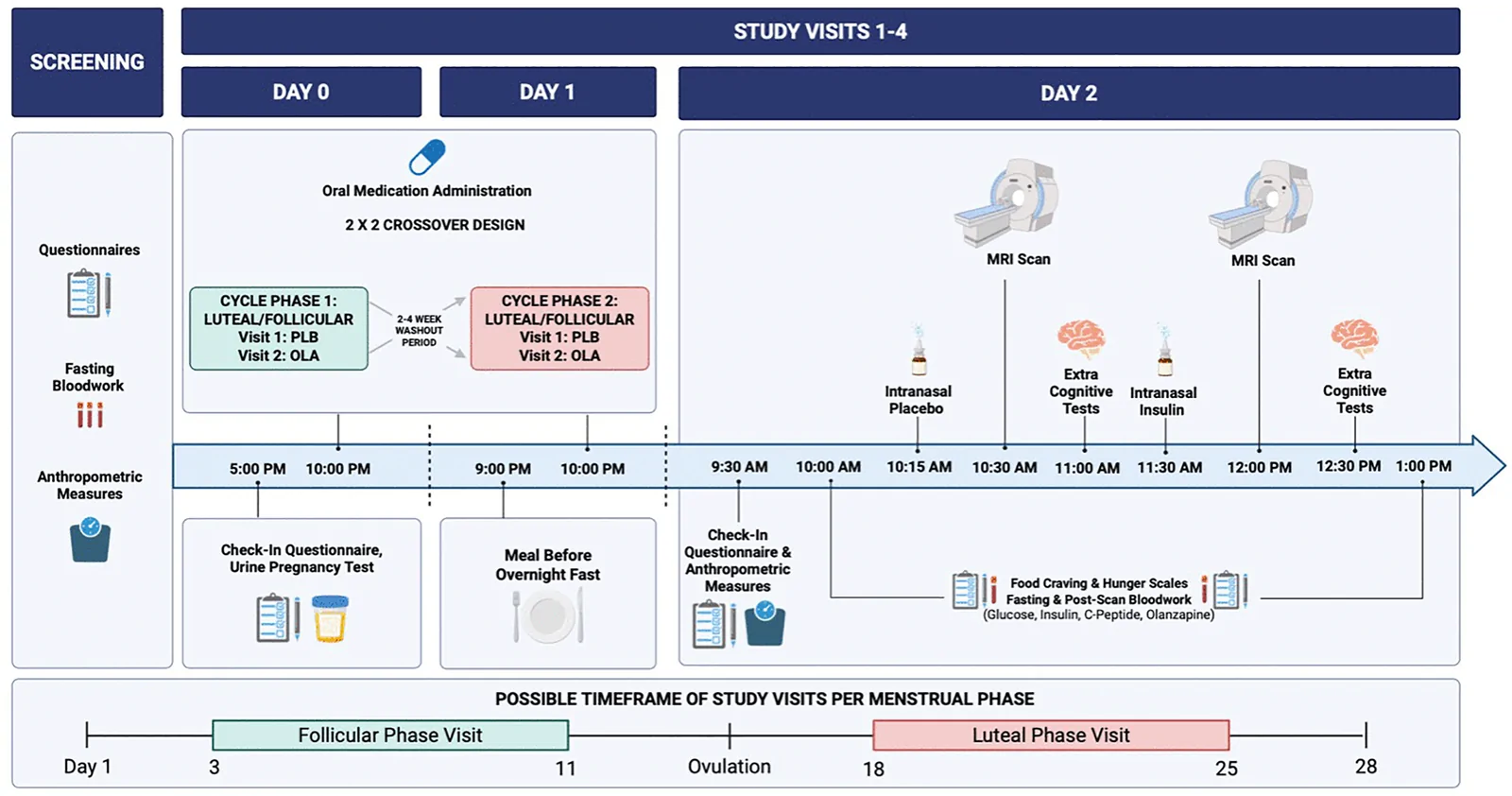
Summary of overall study design and visit protocol. Following screening, eligible participants will be enrolled to take part in the 2x2 crossover study. Each participant will undergo a total of four primary study visits, two per cycle phase (follicular and luteal). Participants will receive oral placebo (PL) on days 0 and 1 of the first visit of each phase, and oral olanzapine (OLA) on days 0 and 1 of the second visit of each phase. At each visit, intranasal placebo (INP) will be administered prior to scan #1, and intranasal insulin (INI) prior to scan #2. Figure created on BioRender.com.

Fig 2 provides a comprehensive overview of the entire study design and protocol. In a single-blind crossover study, every participant is given four different treatment conditions (INP/PL, INI/PL, INP/OLA, or INI/OLA) on two separate occasions. This means that each treatment is administered once during each menstrual cycle phase, resulting in participants receiving all conditions twice over the course of the study. All participants will receive oral PL at visit 1 and oral OLA at visit 2 of each cycle phase. Study visits will take place 2–6 weeks apart to ensure specific menstrual phase across the four visits and adequate washout between conditions. Participants will undergo two visits during the follicular phase of the menstrual cycle. To ensure a regular menstrual cycle, participants will be asked to provide information about the length of their last three cycles. Average cycle duration will be calculated to determine cycle phases. One cycle duration is defined as from cycle onset date (Day 1) to the day before the next cycle onset (i.e., Day 1 is defined as the first day of menstruation). Based on this, participants will be invited to complete the visits during the specific windows of their follicular and luteal phase. Menstrual cycle phases will be identified using calendar-based counting. The onset of their menstruation will be considered as the first day of the current menstrual cycle and will be denoted the start of the follicular phase. The follicular phase visit can take place starting 2 days after the start of the current menstrual cycle and no more than 17 days apart from the start of the subsequent cycle (i.e., they will be scanned between days 3 and 11 of their cycle). The luteal phase will be defined in the time frame of a maximum of 10 days but no less than 3 days before the start of the subsequent cycle (i.e., between day 18–25). Measurement of serum sex hormones (progesterone, estrogen, luteinizing hormone (LH) and follicular stimulating hormone (FSH)) will be done at each visit to confirm cycle phase.

Each of the study periods will involve a three-day protocol (Fig 2): administration of OLA 5 mg (or PL) HS on day 0, OLA 10 mg (or PL) HS on day 1, and cognitive testing and MRI scanning on day 2. There will be two MRI scans on day 2, the first following administration of INP and the second following 160 IU of INI. Administration of INP always precedes INI as the effect of insulin in the brain is known to last for 2 hours, and counterbalancing the administration of INI first will require a much longer washout period/study visit, adding participant burden.

The two acute OLA doses of 5 mg and 10 mg are chosen for reasons of practicality and safety in line with previous studies [24,39]. The INI dose is the maximum safest dose that has been administered in humans and is chosen based on earlier work in nine healthy males showing a dose-dependent relationship on regional brain activity, with the strongest effects being observed after 160 IU [27]. This dose has been shown to induce changes in the brain in females as well [40,41]. Blood samples will be drawn before and after the MRI to measure insulin spillover. Dose-dependent spillover of INI into peripheral circulation has been reported, although the observed brain responses were not correlated with this transient rise in circulating insulin [27]. OLA levels will be measured to assess adherence.

### Investigational products

The oral investigational agent used in this study is olanzapine (Generic brand: Apo-Olanzapine), administered at night over the course of two days (5 mg and 10 mg, respectively). The intranasal investigational product will be either Insulin Lispro (Humalog) or placebo (0.9% saline solution) administered via a metered nasal dispenser. A single spray dispenses 0.1 mL (10 IU) of insulin. A single spray is administered in each nostril while inhaling. After waiting 1 minute, another spray is similarly administered in each nostril. The intranasal spray process in both nostrils is repeated for a total of 8 times (total of 160 IU will be delivered). Medication adherence will be monitored by collecting empty medication blister packs and measuring blood levels of OLA. INI/INP will be administered in the presence of study staff.

### Study participants

This study will recruit fifteen healthy female volunteers aged 18–35 years, of any race or ethnicity, with normal body mass index (BMI 18.5–25 kg/m²) and regular menstrual cycles. Participants will be excluded from the study for the following reasons: 1) History of psychiatric illness (the Mini International Neuropsychiatric Interview (MINI) will be used to screen for psychiatric illness in the present or past), 2) Pre-diabetes or diabetes (fasting glucose ≥6.0 mmol/L, HbA1c>6% or use of any anti-diabetic drug), 3) Evidence of impaired insulin sensitivity, assessed using the Homeostatic Model Assessment for Insulin Resistance (HOMA-IR) ≥1.8 [42,43], 4) Family history of diabetes in a first degree relative (parent or sibling), 5) Use of weight reducing agents, 6) History of kidney or liver disease, 7) Moderate-to-severe substance use, 8) Irregular menstrual cycles (e.g., menstruation occurs less than 21 days or more than 35 days apart, or not having menstruated for three months (or 90 days), or conditions such as endometriosis or polycystic ovary syndrome (PCOS), or prior surgical interventions such as a hysterectomy or oophorectomy), 9) Use of hormonal birth control, 10) Current use of progesterone, estrogen, testosterone, or fertility treatment, 11) Pregnant or breastfeeding, 12) Major medical or surgical event within the last 6 months, 13) Any condition that interferes with safe acquisition of MRI data such as metal implants, pacemakers, cochlear implants, claustrophobia, etc., 14) Any contraindications to the investigational products as listed in the product monographs including known hypersensitivity to the drug or the excipients of the product (note: enzymatic lactose intolerance is NOT exclusionary), 15) Use of any of the prohibited medications listed in the product monograph of olanzapine (e.g., Levodopa and dopamine agonists and antihypertensive agents).

Participants can continue to receive routine medical care while enrolled. Any participant may be discontinued from the study at the discretion of the investigators if this is deemed to be in the best interest of the participant. The decision may be made either to protect the participant’s health and safety, or because it is part of the research plan that people who develop certain conditions may not continue to participate. Reasons for withdrawing individual participants from the study may include one or more of the following: a) major protocol violation, b) participant lost to follow-up, c) withdrawal of consent, or d) participant becomes pregnant or breast-feeding.

### Recruitment and trial procedures

Healthy volunteers will be recruited through approved postings in the community. The CAMH Research Registry will also be used to recruit participants for this clinical trial. Upon REB approval to use the Research Registry as a recruitment strategy, authorized research personnel will search and contact potential research participants included within the member database of the Research Registry for study participation. This clinical trial will also be posted on the Research Registry website, as well as the public CAMH website. Once posted, interested participants can use the “Find a CAMH study” feature to explore clinical trials that they are interested in.

Interested individuals will be pre-screened by study staff with the REB approved “pre-screen form” to determine preliminary eligibility. If deemed eligible based on this form, participants will be invited to complete informed consent and the begin the official screening procedures of the study. Study staff will obtain written, signed informed consent from each participant. The study team will maintain records for the number of people approached, screened, eligible, withdrawn, and the reasons for non-enrollment. Any research information recorded for, or resulting from, participation in this research study prior to the date that the participant formally withdrew their consent will be retained and may continue to be used and disclosed by the investigators for research purposes; however, no new data will be collected.

### Randomization, blinding, and allocation concealment methods

Study staff will confirm eligibility and assign each participant a unique trial number. As all participants will receive oral PL at visit 1 and oral OLA at visit 2 of each cycle phase, no randomization of investigational products will occur. Study staff will remain unblinded to ensure accurate scheduling of visits during each phase. Both INP and INI will be administered at every MRI visit: INP will always be delivered before the first scan and INI before the second scan. Participants will be blinded to the order of these interventions and will not know whether they have received OLA or PL. Oral OLA and PL will be dispensed by the pharmacy in identical capsules packaged in blister packs. INI and INP will be transferred from their respective vials into identical, unlabeled metered spray bottles to maintain participant blinding.

### Study assessments

#### Screening visit.

Prior to inviting participants to complete the consent form and begin the screening visits of the study, participants will be asked a series of eligibility-related questions through a telephone pre-screen to act as an initial check of eligibility and safety.If the participant has been deemed eligible, they will proceed to the screening visits of the study for informed consent and thorough assessment of eligibility. Screening will begin with obtaining informed consent, followed by a review of each participant’s medical history and demographic information. Participants will undergo fasting bloodwork, including fasting glucose, insulin, c-peptide, lipid profile, HbA1c, complete blood count, prolactin, vitamin B12, electrolytes, thyroid function, and liver and kidney function tests. Anthropometric measures will also be collected, including height, weight, blood pressure, and waist circumference. Edinburgh Handedness Inventory (EHI) will be used to assess hand dominance or laterality [44]. Psychiatric screening will be conducted using the Mini-International Neuropsychiatric Interview (MINI) [45] to exclude individuals with mental health disorders. To estimate menstrual phases for visit schedule, participants will be asked to report their last three menstruation dates to determine average cycle length. Progesterone, estrogen, LH, and FSH levels obtained through bloodwork will be used to confirm cycle phase (follicular phase defined by high estrogen; luteal phase defined by high progesterone). All pre-screening and screening assessments will be performed by trained study stuff and reviewed by the study PI for eligibility. Participants will only be enrolled in the study once eligibility has been determined following the screening visit.

#### Study visits 1–4.

Each study visit follows an identical three-day protocol. Approximately one week before the schedule visit, study staff will contact the participant to confirm that there have been no changes in health status and that menstrual cycles remain regular. Participants will be asked to provide the date of their most recent menstrual period to ensure that the visit falls within their assigned follicular or luteal phase. They will also be reminded to abstain from recreational drug use during the week leading up to the MRI session. The three-day protocol will be as follows:

Day 0: Participants will undergo a pregnancy test and will be instructed to take 5 mg of oral OLA (or PL) at night at their home between 9:30 and 11:30 pm.

Day 1: Participants will receive a standardized mixed meal (with vegan and vegetarian options) to be consumed at their usual dinner time or no later than 9:30 pm. This will serve as their final meal of the day, after which they must remain fasted until completing the study visit the next day. On the night of Day 1, participants will take 10 mg of oral OLA (or PL) before bed between 9:30–11:30 pm.

Day 2: Participants will be picked up by taxi from their home and brought to CAMH for the primary study assessments to occur. Participants will first undergo fasting bloodwork to measure glucose, insulin, c-peptide, estrogen, progesterone, LH, FSH, and OLA levels. Blood glucose, insulin, and c-peptide will be measured again after the MRI session. The visit includes two MRI scans: INP will be administered before MRI Scan #1 (approximately 30 minutes), followed by INI prior to MRI Scan #2 (approximately 30 minutes). MRI will be performed using CAMH’s research dedicated 3.0 Tesla GE scanner equipped with a 32-channel head coil. The scanning protocol will include a high-resolution T1-weighted structural image and a functional MRI (fMRI) scan to measure resting state brain activity.

Cognitive assessments will be conducted following the administration of both INP and INI, and will include the Brief Visuospatial Memory Test–Revised from the Kaplan Functional System [46] as well as the Digit Symbol Substitution Test [47] Participants will also complete a series of questionnaires and rating scales, including the 3-day food diary, the Food Craving Scales, the Profile of Mood States (POMS) [48], the Subjective Wellbeing Under Neuroleptics (SWN) [49], the Barnes Akathisia Rating Scale (BAS) [50], and the Stanford Sleepiness Scale (SSS) [51]. Anthropometric measures will also be collected, including height, weight, waist circumference, and blood pressure.

The above procedures are repeated for a total of 4 research visits (two of which will occur during the follicular phase and two during the luteal phase).

### Data analysis

#### MRI analysis.

The rsFC analyses, including pre-processing and denoising, will be conducted using a seed ROI-to-ROI approach implemented in the CONN toolbox (version 18b; https://www.nitrc.org/projects/conn). Functional images will be slice-time and motion corrected, realigned, co-registered to structural scans, normalized to MNI-space, and spatially smoothed with an 8-mm FWHM Gaussian kernel.

### Statistical analysis

The primary outcome is rsFC. Given the exploratory nature of this study, a formal power calculation was not performed. The sample size (N = 15) was instead informed by prior neuroimaging studies examining the effects of INI on MRI-derived outcomes, which have which have typically included 9–25 participants and reported large effect sizes (Cohen’s d: 0.8 to 1.4, f: 0.4–0.6). In the context of a within-subject crossover design, which reduces between-subject variability and increases statistical efficiency, this sample size is expected to provide adequate sensitivity to detect moderate-to-large effects in resting-state functional connectivity.

To address hypothesis #1, which is the difference in brain insulin sensitivity between cycle phases, analyses will be restricted to visits in which participants receive the oral PL. A linear mixed effects model will be conducted with rsFC as the dependent variable and fixed effects for intranasal condition (i.e., INI vs. INP), menstrual phase (follicular vs. luteal), and their interaction. Participant will be included as a random intercept, with age and BMI entered as covariates. The primary effect of interest is the intranasal condition x menstrual phase interaction, which tests whether the effect of INI (relative to INP) differs between follicular and luteal phases. Planned simple effects will assess (i) the effect of INI vs. INP within each phase, and (ii) the difference in INI-induced change (INI – INP) between phases.

To test the effect of OLA within each phase, a second linear mixed effects model will be conducted including all visits. Fixed effects will include intranasal condition (INI vs. INP), oral condition (OLA vs. PL), menstrual phase (follicular vs. luteal) and all interaction terms. Participants will be modeled as a random intercept, with age and BMI as covariates. The primary term of interest is the intranasal condition x oral condition x menstrual phase interaction, which tests whether OLA modifies INI-induced effects differently across cycle phases.

The primary analysis will be conducted according to the intention-to-treat (ITT) principle, including all participants enrolled in the study who have completed at least one study visit. This analysis will account for missing data under the missing at random (MAR) assumption using the maximum likelihood estimation within the linear mixed model’s framework. A per-protocol analysis will also be conducted as a sensitivity analysis, including only participants who completed the study with full adherence to the protocol, to assess the robustness of the primary findings.

All statistical analyses will be conducted using IBM SPSS Statistics Version 31 or R 4.3.2 (R Foundation for Statistical Computing, Vienna, Austria). The level of statistical significance will be set at α = 0.05 for all primary analyses, with corresponding confidence intervals reported where applicable. Adjustment for multiple comparisons will be applied as appropriate, using either Bonferroni correction or false discovery rate (FDR).

### Assessment of safety

All adverse events will be recorded in the Adverse Event Log. Adverse events include any unfavorable change in a study participant’s physical or psychological wellbeing during the study period, which may or may not be the result of participation in the study. Adverse events will be reported to the CAMH REB if all of the following REB-defined criteria are met for an Unanticipated Problem: 1) The event is unexpected (relative to the product monograph, research protocol, and consent forms), 2) The event is related or possibly related to participation in the research, as judged by the PI, 3) The event suggests that the research places the participants or others at a greater risk of harm than was previously known or recognized. Required reporting will occur within 48 hours of the PI becoming aware of the event. Any Serious Unexpected Adverse Drug Reactions will be reported to Health Canada if all of the Health Canada-defined criteria are met: 1) The medical occurrence is serious (results in death, is life-threatening, requires hospitalization or prolongation of existing hospitalization, or is a congenital anomaly/birth defect), 2) The event is unexpected (nature or severity not consistent with information in the relevant source documents such as product monograph), and 3) It is judged by the reporting healthcare professional as a having a reasonable suspected causal relationship to the medicinal product. Where the event is neither fatal nor life threatening, required reporting will occur within 15 days of awareness of the information. If fatal or life-threatening, it will be reported within 7 days.

### Confidentiality

There is a potential risk of breach of confidentiality that is inherent in all research protocols. Breach of confidentiality will be minimized by the staff who will maintain research data (identified only by participant code number not related to name, or date of birth) in separate charts and a dedicated password protected electronic database. A list of participant names, their ID numbers, and information about how they can be reached will be kept in a separate locked cabinet with access only to study personnel authorized by the PI. Procedures have been established, and will be followed, to minimize the risk of breach of confidentiality. Procedures to maintain confidentially include: (1) formal training sessions for all research staff emphasizing the importance of confidentiality; (2) specific procedures developed to protect participants’ confidentiality; and (3) formal mechanisms limiting access to information that can link data to individual participants. All information obtained from participants will be kept as confidential as possible. Computer-based files/data will be entered into password-secured databases and paper-based files will be stored in a secure location. These data will only be accessible to personnel involved in the study and they will abide by confidentiality regulations of the REB. The REB will be granted direct access to the study participants’ original medical records for verification of clinical trial procedures and/or data, without violating the confidentiality of the participants, to the extent permitted by the law and regulations.

### Study management and materials

CAMH investigators will retain a participant identification code list if they need to contact participants after the study. This list will contain the complete name, identification number, address and phone number of all participants and will be held confidentially at the investigators site after completion of the study. Study data will be entered in a secure database. An eCRF/CRF will be completed for each participant enrolled in the study. A participant screening log, noting reasons for screen failure, where applicable, will be maintained for all participants. The investigator will document the obtained informed consent and record medical and psychiatric history, medications, and efficacy data in the eCRF/CRF. Clinical scales and neuropsychological assessments will be considered source documents and will be incorporated into the eCRF/CRF in a confidential manner. Study data will be entered in a secure database using Research Electronic Data Capture (REDCap) software. At point-of-entry, data values will undergo consistency edits (e.g., ID validation, range verification, duplicate detection) and personnel will be required to correct errors.

### Recruitment status and study timeline

Recruitment for this study began in May 2024 and has been ongoing ever since. At the time of this submission, a total of 25 participants have consented to the study, 13 of which have been eligible and enrolled. Three participants dropped out at various stages of their participation, six have completed their full participation, and the remaining are still actively enrolled and completing study visits. The retention and successful completion of participants speak to the feasibility of the study being conducted in this population. Recruitment is currently ongoing and is anticipated to be completed by June 2026. Results and an associated publication are expected 6–12 months following trial completion.

### Knowledge transmission and dissemination plans

The results from the current study will be presented at international conferences and published in open access journals to ensure widest reach to other qualified researchers and clinicians.

## Discussion

Females suffer the most from AP-related metabolic adverse effects. Brain insulin action is increasingly being recognized as a potential mediator of these side effects. Disappointingly, females are an underrepresented population in the field because of the complex confounding effects of monthly hormonal variations. This is the first study to explore the effect of menstrual cycle phases on the anti-insulin action of APs. Thus, this investigation has the potential to generate new knowledge in an area of significant unmet need. Demonstrating that APs disrupt brain insulin action, evidenced by inhibition of recognized effects of INI on neuroimaging measures, will provide novel insights into currently poorly understood mechanisms. Moreover, it may provide us with a modifiable risk factor that can then be targeted using brain insulin sensitizers to address the metabolic dysfunction (glucose metabolism, satiety, food choice) and possibly cognitive deficits at the earliest stages of the illness. This approach can improve the quality of life, long-term outcomes, and overall health of females suffering from SSD and its treatment consequences.

### Limitations and measures to minimize bias

It is recognized that the single-blind nature of this study is a limitation but required to ensure the proper distribution of study conditions across cycle phases. The trial is registered with a publicly available, free-to-access, searchable clinical trial registry before approaching the first participant to avoid bias selection of the reported result(s). Furthermore, study staff administering and scoring the extra-scanner cognitive assessments will be blinded to the INI/INP allocation to minimize any bias (they will not be involved in transferring the product into the nasal spray bottle).

Participants may experience mild subjective effects related to the study interventions (e.g., nasal irritation with intranasal administration or transient sedation with OLA), which could potentially compromise the single-blind design. To mitigate this, placebo conditions will be matched as closely as possible in appearance and administration, and participants will remain unaware of treatment allocation. Furthermore, a questionnaire will be completed at each visit to assess participant blinding success. Participants will be asked whether they believe they received the OLA or PL at that visit, as well as whether they received INP or INI they received prior to each MRI scan, and to rate their confidence in these judgements. The James’ Blinding Index (BI) will be calculated from these responses [52].

## Supporting information

S1 File REB clinical study protocol. (DOCX)

S2 File SPIRIT checklist. (DOC)
