# Supplementary material for: Effects of antipsychotics on brain insulin action in females: Study protocol for a placebo-controlled, crossover neuroimaging study
Source: PLoS One. 2026 Aug 27;21(8):e0352262. doi: 10.1371/journal.pone.0352262 (PMC13521361; doi:10.1371/journal.pone.0352262)
Supplement: Supplementary file 1 [file pone.0352262.s001.docx]

**Effects of antipsychotics on brain insulin action in females: a placebo-controlled, crossover multi-modal neuroimaging study**

**Protocol Identifying Number: *127/2023***

**Version Number: *5.0***

**Version Date: *11 Oct 2024***

**Table of Contents**

**STATEMENT OF COMPLIANCE**	5

**LIST OF ABBREVIATIONS	6**

**CLINICAL TRIAL SUMMARY	7**

**1.0	INTRODUCTION**	9

1.1	Background	9

1.2	Study Intervention	11

1.3	Preclinical Data to Date	11

1.4	Clinical Data to Date	12

1.5	Risks/Benefits	12

**2.0	CLINICAL TRIAL OBJECTIVES**	13

2.1	Primary Objective	13

2.2	Secondary Objective	13

**3.0	CLINICAL TRIAL DESIGN**	13

3.1	Overall Design	13

3.2	Primary Endpoints	14

3.3	Secondary Endpoints	14

**4.0	PARTICIPANT SELECTION AND WITHDRAWAL**	14

4.1	Target Population	14

4.2	Participant Recruitment and Screening	15

4.3	Equity, Diversity and Inclusion Considerations	16

4.4	Eligibility Criteria	16

4.4.1	Inclusion Criteria	16

4.4.2	Exclusion Criteria	17

4.5	Lifestyle Considerations	17

4.6	Screen Failures	18

4.7	Participant Withdrawal Criteria	18

4.7.1	When and How to Withdraw Participants	18

4.7.2	Follow-up for Withdrawn Participants	18

**5.0	STUDY INTERVENTION**	19

5.1	Description	19

5.2	Treatment Regimen	20

5.3	Method for Assigning Participants to Treatment Groups	20

5.4	Administration of Study Intervention	20

5.5	Participant Compliance Monitoring	21

5.6	Concomitant Therapy	21

5.7	Packaging	22

5.8	Blinding of Study Intervention	22

5.9	Receiving, Storage, Dispensing and Return	22

5.9.1	Receipt of Study Intervention Supplies	22

5.9.2	Storage	22

5.9.3	Dispensing of Study Intervention	23

5.9.4	Return or Destruction of Study Intervention	23

**6.0	RESEARCH PROCEDURES**	23

6.1	Research Visits	23

6.2	Schedule of Events	25

**7.0	STATISTICAL PLAN**	27

7.1	Sample Size Determination	27

7.2	Statistical Methods	27

**8.0	SAFETY AND ADVERSE EVENTS**	27

8.1	Definitions	27

8.2	Recording of Adverse Events active language	29

8.3	Reporting of Serious Adverse Events	29

8.3.1	Investigator Reporting: Notifying the Sponsor	29

8.3.2	Investigator Reporting: Notifying the REB	29

8.3.3	Sponsor Reporting of SUADRs: Notifying Health Canada	29

8.3.4	Sponsor Reporting of SUADRs: Notifying Sites	30

8.4	Reporting of Device Deficiencies	30

8.5	Safety Management Plan	30

8.6	Unblinding Procedures	30

8.7	Data and Safety Monitoring Board	30

**9.0	CLINICAL TRIAL DISCONTINUATION AND CLOSURE**	31

9.1	Clinical Trial Discontinuation	31

10.0	DATA HANDLING AND RECORD KEEPING	31

10.1	Source Documents & Case Report Forms	31

10.2	Protocol Deviations	31

10.3	Record Retention	31

10.4	Clinical Trial Registration	31

11.0	STUDY MONITORING, AUDITING, AND INSPECTING	32

11.1	Study Monitoring Plan	32

11.2	Auditing and Inspecting	32

**12.0	ETHICAL CONSIDERATIONS**	32

12.1	Research Ethics Board (REB) Approval	33

12.2	Informed Consent Process & Documentation	33

**13.0	PRIVACY AND CONFIDENTIALITY**	33

**14.0	CLINICAL TRIAL FINANCES**	35

14.1	Funding Source	35

14.2	Conflict of Interest	35

**15.0	PUBLICATION POLICY/DATA SHARING**	35

15.1	Future Secondary Use of Data	35

**16.0	REFERENCES**	36

# STATEMENT OF COMPLIANCE

This clinical trial will be carried out in accordance with the following:

- International Conference on Harmonisation Good Clinical Practice (ICH GCP)
- Tri-Council Policy Statement 2018 (TCPS 2)
- Personal Health Information Protection Act (PHIPA), 2004; Chapter 3 Schedule A (PHIPA) and applicable regulations
- Food and Drugs Act
  - Part C, Division 5 of the Food and Drug Regulations
  - Part 3, Medical Device Regulations
  - Part 4, Natural Health Products Regulations
  - Institutional and REB policies and procedures

________________________________	_____________________________

Signature of PI	Date

*or*

Signature of site PI *(multi-centre clinical trials)*

# LIST OF ABBREVIATIONS

*AE	Adverse Event*

*ASL	Arterial Spin Labeling*

*BMI	Body mass index*

*CRF	Case report form(s)*

*DSMB	Data Safety & Monitoring Board*

*DTA*	*Data Transfer Agreement*

*ASL*	*Arterial Spin Labeling*

*fMRI*	*Functional MRI*

*INI*	*Intranasal Insulin*

*INP*	*Intranasal Placebo*

*OLA*	*Olanzapine*

*PL*	*Placebo*

*GCP	Good Clinical Practice*

*ICF	Informed consent form*

*MRI	Magnetic resonance imaging*

*MRS*	*Magnetic resonance spectroscopy*

*ICV*	*Intracerebroventricular*

*PHI	Personal Health Information*

*PHIPA	Personal Health Information Protection Act*

*PI	Principal Investigator*

*QI*	*Qualified Investigator*

*SAE	Serious Adverse Event*

*SUSAR	Suspected unexpected serious adverse reaction*

*TCPS 2*	*Tri-Council Policy Statement*

# CLINICAL TRIAL SUMMARY

| **Title** | *Effects of antipsychotics on brain insulin action in females: a randomised placebo-controlled, crossover multi-modal neuroimaging study* |
| --- | --- |
| **Short Title** | *Brain insulin resistance in females* |
| **Phase** | *N/A* |
| **Methodology** | *Single-blind crossover design* |
| **Clinical trial Duration** | *24 months* |
| **Participating site(s)** | *Single-Centre study: Centre for Addiction and Mental Health (CAMH)* |
| **Objectives** | *This study will investigate; how brain insulin action differs in females according to their menstrual cycle phase, and how a high metabolic liability agent such as OLA might interrupt these differential insulin effects* |
| **Number of Participants** | *15* |
| **Study Intervention**  **Reference Therapy/Comparator** | *All participants will complete an MRI-based protocol where each one receives 4 different treatment combinations (INP/PL, INI/PL, INP/OLA or INI-OLA) twice over 4 separate occasions.* |
| **Duration of Intervention** | *Study visits will take place 2-6 weeks apart to ensure specific menstrual phase across all visits.* Participants will be enrolled in the study for up to 5-6 months. |
| **Statistical Methodology** | *Mixed Effect Models will be used to investigate the impact of menstrual phase on the effect of INI in females, and the additive effect of OLA on this response. The four treatment conditions, group (i.e., follicular phase vs. luteal phase), and time, will be entered as predictors and participants will be used as random factors in a random intercept model. Age, BMI, and sedation levels (due to OLA intake) will be used as covariates in the analysis. Additionally, these data will be*  *compared with the subgroup of male participants from our previous study with the exact same inclusion criteria and imaging acquisition parameters to examine sex effects.* |

# 1.0 INTRODUCTION

## 1.1 Background

Antipsychotic use and metabolic adverse effects: Antipsychotic (AP) medications are the cornerstone of treatment for schizophrenia (SCZ) spectrum disorders ^1^ and are increasingly prescribed off-label and among children ^2^. Unfortunately, their use is associated with serious metabolic adverse effects including weight gain, glucose dysregulation, type 2 diabetes, and cardiovascular disease ^3–8^, contributing to a 15-20-year shorter lifespan than the general population ^9^. Furthermore, these adverse effects accrue early in the course of treatment; alarmingly high rates of dyslipidemia (>55%) and prediabetes (>15%) have been reported in first episode patients within 6 months of AP exposure ^10^. Of particular concern is that females are disproportionally more affected by these adverse effects than males ^11,12^; findings from the CATIE study revealed that the prevalence of metabolic syndrome in patients with SCZ was 51.6% among females compared to 36% among males ^13^. AP associated metabolic disturbances are linked to non-adherence, lower self-esteem, and decreased quality of life ^14^, and might exacerbate pre-existing cognitive dysfunction in SCZ ^15^. These cumulative effects significantly increase the illness burden of SCZ. Notably, there is evidence to suggest that females with SCZ tend to report lower quality of life than males with SCZ, despite having less severe course of the illness ^16^. It is possible their increased susceptibility for the metabolic side effects of APs may contribute to the reported lower quality of life than males.

Mechanisms underlying glucose dysregulation with antipsychotics: Although the pathophysiology of glucose dysregulation with APs is largely undetermined, direct weight-independent effects of APs on glucose regulating mechanisms has received recent attention ^17–19^. Perturbations in glucose regulation may be the earliest sign of metabolic disturbance. Indeed, pre-clinical data in male rodents have shown that single doses of AP reliably cause acute, profound perturbations in glucose metabolism at the level of the liver ^20,21^. Similar findings are reported in humans (in both sexes), where 1-9 days of olanzapine induced significant changes in glucose metabolism, with no associated adiposity changes ^22,23^. Interestingly, the brain is believed to be a potential modulator of these effects ^24^.

The role of insulin in the brain: It has recently been hypothesized that abnormalities in brain insulin action may underlie the increased risk of type 2 diabetes in SCZ ^25,26^. Insulin receptors are widely expressed in the brain and bear on multiple physiologic processes ^25^. For instance, insulin, separate from its effects in the periphery, regulates hepatic glucose production through insulin receptors in the hypothalamus. Using the pancreatic euglycemic clamp technique to separate central and peripheral insulin effects, it has been established that an intracerebroventricular (ICV) infusion of insulin in male rodents results in a decrease in glucose production by the liver ^27,28^. Similar findings have been observed in healthy male humans with intranasal insulin (INI) (which is a direct, non-invasive way of delivering insulin to the brain, analogous to ICV infusions in rodents) ^29^.

Disruption of brain insulin action with antipsychotics: Interestingly, our group has shown that an acute dose of olanzapine (OLA) can completely abolish this well-established ability of a central insulin stimulus to decrease hepatic glucose production in male rats ^30^. This suggests that APs have a direct anti-insulin action in the brain that is independent of weight gain. Despite reported sex differences in the effects of brain insulin on energy balance in the literature where males are believed to be more sensitive to the catabolic actions of insulin than females, ^31,32^ this finding is yet to be explored in females. Preclinical evidence has demonstrated that ICV infusion of insulin decreases food intake and body weight in male rats but not female rats, male rats given estradiol, or ovariectomized female rats ^33^. This suggests that insulin action in the brain is sex-dependent and requires further investigation.

Assessing brain insulin action in humans using neuroimaging: Neuroimaging studies have employed INI to demonstrate the effects of insulin on the brain. In a study including both sexes, INI enhanced connectivity in the default-mode network (DMN), measured using resting state functional magnetic resonance imaging (rs-fMRI) ^34^. Arterial spin labeling (ASL) studies have shown that INI reduces cerebral blood flow in the hypothalamus and prefrontal cortex (PFC) in healthy males ^35^, and increases perfusion in the insular cortex in both sexes ^36^. Lastly, proton magnetic resonance spectroscopy (MRS) showed that infusion of insulin in healthy males increases glutamate levels in the frontal and temporal cortex ^37^, but only in participants who were not insulin resistant. In fact, insulin resistance has been correlated with lower striatal glutamate ^38^. This finding is important as APs are known to decrease striatal glutamate ^39^, which is otherwise high in unmedicated first episode SCZ ^40^. This ability of INI to induce changes in MRI-based parameters in healthy humans allows it to be leveraged as an assay of insulin action in the brain. Lack of expected responses to an INI challenge is interpreted as a lack of insulin sensitivity. However, these results are primarily based on findings in studies in males, with only a limited number of studies examining females only or both sexes.

Studying AP-induced disruptions on brain insulin action in humans using MRI: In a proof-of concept study at CAMH, we used INI and multi-modality MRI to investigate whether an acute dose of olanzapine can induce insulin resistance in the brain in healthy males and females ^41^. Twenty-two participants (N=11 females) with no psychiatric illness or metabolic dysfunction completed the study. In line with our hypotheses, we observed that INI induced changes in cerebral blood flow within the right dorsal anterior cingulate cortex (**Figure 1**), which was attenuated by olanzapine. However, an interesting finding is that these effects were only observed in the male participants, but not females (**Figure 1**). This suggests a sex difference in brain insulin action.

**Figure 1**: Regional cerebral blood flow (rCBF) in the right dorsal anterior cingulate cortex (ACC). INI-induced change and its attenuation with olanzapine is seen in male participants only (middle panel). INP: Intranasal Placebo; INI: Intranasal Insulin; PL: oral placebo; OLA: oral olanzapine

Sex Differences in Insulin Sensitivity: As alluded to previously, there continues to be a significant sex bias in our understanding of insulin effects in the brain as most of the research with INI has been conducted in males alone ^29,35,42–46^. In the few studies that have included both males and females, differential effects according to sex have been noted. For example, INI appears to acutely reduce food intake in fasted, normal-weight males, but not females ^47^; however, INI has been shown to increase satiety and reduce the palatability of certain foods in females ^48^. Similarly, 8 weeks of INI treatment resulted in a significant reduction in body weight and body fat content in males only ^32^.

A recent report (published after we initiated our pilot study) has indicated that brain insulin sensitivity differs between sexes, and this may relate to menstrual phases ^49^. Specifically, brain insulin sensitivity in females is higher and comparable to lean males during the follicular phase (i.e., right before ovulation when estrogen levels are high) than during the luteal phase (i.e., after ovulation when both estrogen and progesterone levels are high) ^49^, and this may contribute to the well-established decrease in whole-body insulin sensitivity in the luteal phase ^50,51^.

There has yet to be a study that investigates AP-induced insulin resistance in females and the impact of menstrual cycle factors on this relationship. Preclinical evidence of AP-induced disruption in brain insulin action was in male rats only, and our previous neuroimaging study aimed at translating these findings did not investigate the effect of menstrual phase. It is therefore critical to study the effect of APs on brain insulin action in females, and to examine how it fluctuates with the menstrual cycle. ^52^

## 1.2 Study Intervention

All participants will complete a magnetic resonance imaging (MRI)-based protocol where each participant will receive 4 different treatment conditions (INP/PL, INI/PL, INP/OLA or INI-OLA) twice over four separate occasions.

## 1.3 Preclinical Data to Date

A preclinical study has shown that an acute dose of olanzapine (OLA) can completely abolish this well-established ability of a central insulin stimulus to decrease hepatic glucose production in male rats ^30^. Another preclinical study demonstrated that ICV infusion of insulin decreases food intake and body weight in male rats but not female rats, male rats given estradiol, or ovariectomized female rats ^33^. This suggests that insulin action in the brain is sex-dependent and requires further investigation.

## 1.4 Clinical Data to Date

In a study including both sexes, INI enhanced connectivity in the default-mode network (DMN), measured using resting state functional magnetic resonance imaging (rs-fMRI) ^34^. Arterial spin labeling (ASL) studies have shown that INI reduces cerebral blood flow in the hypothalamus and prefrontal cortex (PFC) in healthy males ^35^, and increases perfusion in the insular cortex in both sexes ^36^. Lastly, proton magnetic resonance spectroscopy (MRS) showed that infusion of insulin in healthy males increases glutamate levels in the frontal and temporal cortex ^37^, but only in participants who were not insulin resistant. In fact, insulin resistance has been correlated with lower striatal glutamate ^38^. This finding is important as APs are known to decrease striatal glutamate ^39^, which is otherwise high in unmedicated first episode SCZ ^40^.

## 1.5 Risks/Benefits

Blood draws for most people do not cause any serious problems. However, they may cause minor discomfort, bleeding, bruising, soreness or infections at the site on rare occasions.

There are no known lasting effects associated with MRI scanning. Participants may perceive minor discomfort due to the scanner sounds. They will be provided with earplugs to minimize discomfort. Participants will be screened thoroughly to ensure that there are no metallic objects or implants on them.

Some of the assessments, in particular, the questionnaires, may be upsetting to study participants. We will minimize all of these by having all assessments performed by well-trained research staff with clinical experience and appropriate skills to maximize the participants’ comfort and keep their distress to a minimum during each visit.

Olanzapine is approved for the treatment of psychotic illness. It can cause side effects such as fatigue/sleepiness (26%), headache (17%), dry mouth (7%), constipation (9%), and muscle side effects. Muscle side effects may occur, and include restlessness (23%), decreased movements (<5%), muscle stiffness and rigidity (<5%) or abnormal posture of neck or hand due to muscle rigidity, called dystonia (<5%). If any of these side effects occur, they will be assessed and treated promptly.

In humans, with acute administration of intranasal insulin, no adverse effects have been reported. Moreover, in more chronic studies, 8 weeks of intranasal administration of intranasal insulin at a dose of 40 IU QID (total daily dose 160 IU) did not cause any adverse effects.

In general, placebo medication does not cause any side effects. All these side effects are temporary and will disappear (if they occur) within 24 hours of the last dose. A physician will be available at all times during the study to promptly assess and treat any side effects.

# 2.0 CLINICAL TRIAL OBJECTIVES

## 2.1 Primary Objective

To measure brain insulin action in females according to their menstrual cycle phase (resting state functional activity and connectivity).

## 2.2 Secondary Objective

To examine impacts of a high metabolic liability agent such as OLA on the differential insulin effects across menstrual cycle.

# 3.0 CLINICAL TRIAL DESIGN

## 3.1 Overall Design

In a single blind, crossover design, each participant will receive 4 treatment conditions (INP/PL, INI/PL, INP/OLA or INI/OLA) twice on two separate occasions (i.e., the participant will receive each condition once during each cycle phase, twice in total throughout the entire study). All participants will receive oral placebo at visit 1 and oral olanzapine at visit 2 of each cycle phase. There will be a minimum of two weeks between any two visits to ensure adequate washout between conditions.
At each visit, the participant will receive either oral placebo or oral OLA. Each visit will have two MRI scans, the first following INP administration, and the second following INI administration. Study visits will take place 2-6 weeks apart to ensure specific menstrual phase across the four visits. Participants will undergo two visits during the follicular phase of the menstrual cycle. To ensure a regular menstrual cycle, participants will be asked to provide information about the length of their last three cycles. Average cycle duration will be calculated to determine cycle phases. One cycle duration is defined as from cycle onset date (Day 1) to the day before the next cycle onset (i.e., Day 1 is defined as the first day of menstruation). Based on this, participants will be invited to complete the visits during the specific windows of their follicular and luteal phase. Menstrual cycle phases will be identified using calendar-based counting. The onset of their menstruation will be considered as the first day of the current menstrual cycle and will be denoted the start of the follicular phase. The follicular phase visit can take place starting 2 days after the start of the current menstrual cycle and no more than 17 days apart from the start of the subsequent cycle (i.e., they will be scanned between days 3 and 11 of their cycle). The luteal phase will be defined in the time frame of a maximum of 10 days but no less than 3 days before the start of the subsequent cycle (i.e., between day 18-25) ^52^. This is summarized in **Figure 2**. Measurement of serum sex hormones (progesterone, estrogen, luteinizing hormone (LH) and follicular stimulating hormone (FSH)) will be done at each visit to confirm cycle phase.

**Figure 2**. Distribution of study visits across menstrual cycle.

Each of the study periods will involve administration of OLA 5 mg HS (or PL) on day 0, OLA 10 mg HS (or PL) on day 1, and cognitive testing and MRI scanning on day 2. MRI assessments will occur 15 minutes after administering 160 International Units (IU) INI/INP. We have chosen to 1) use OLA as the representative AP given its high metabolic liability, 2) study healthy volunteers to avoid confounding effects of psychiatric illness on glucose metabolism, and 3) utilize acute AP dosing to avoid adiposity changes, allowing us to dissect whether APs directly inhibit brain insulin action. The two acute OLA doses of 5 mg and 10 mg are chosen for reasons of practicality and safety in line with previous studies ^22,41^. The INI dose is the maximum safest dose that has been administered in humans and is chosen based on earlier work in nine healthy males showing a dose-dependent relationship on regional brain activity, with the strongest effects being observed after 160 IU ^35^. This dose has been shown to induce changes in the brain in females as well ^53,54^. Blood samples will be drawn before and after the MRI to measure insulin spillover. Dose-dependent spillover of INI into peripheral circulation has been reported, although the observed brain responses were not correlated with this transient rise in circulating insulin ^35^. OLA levels will be measured to assess compliance.

**MRI Assessments**: MRI will be performed using CAMH’s research dedicated 3.0 Tesla GE scanner equipped with an 32-channel head coil. The scanning protocol will include a high-resolution structural image (~4.5 minutes) and functional MRI (fMRI) to measure the brains activity at rest (~10 minutes). Two fMRI scans will be acquired (one after INP administration and one after INI administration). Image acquisition parameters are identical to those described in the protocol of our previous study ^41^.

**Cognitive Testing**: A brief battery of parallel versions of the Brief Visuospatial Memory Test Revised of the Kaplan Function System ^55^ will be completed in between MRI scans, in line with our previous study ^41^. Cognitive procedures will be completed within the 2 hours available after INI administration because of insulin pharmacokinetics ^36^. Sedation from OLA will be quantified using: a) the Stanford Sleepiness Scale (SSS) ^56^; and, b) the digit symbol substitution test (DSST) ^57^. Any effects of OLA on mood and motivation will be assessed using the Subjective Wellbeing on Neuroleptics (SWN) scale and the Profile of Mood State (POMS) scale.

## 3.2 Primary Endpoints

The primary endpoint of the study is resting state activity and functional connectivity as assessed through a 10-minute resting-state fMRI scanning protocol.

## 3.3 Secondary Endpoints

The secondary endpoints of the study are other cognitive outcomes, including the BVMT and DSST.

# 4.0 PARTICIPANT SELECTION AND WITHDRAWAL

## 4.1 Target Population

Healthy, normal weight female volunteers between the ages of 18 and 35 with a regular menstrual cycle and no history of psychiatric illness or type 2 diabetes. Participants will be screened for eligibility prior to being enrolled in the study. Data from a group of matched healthy males will be taken from another study with the same study protocol (REB #075/2017).

## 4.2 Participant Recruitment and Screening

Fifteen healthy normal weight (BMI 18.5-24.9 kg/m^2^), normal cycling female volunteers between the ages of 18-35 years will be recruited through approved postings in the community. The CAMH Research Registry will also be used to recruit participants for this clinical trial. Upon REB approval to use the Research Registry as a recruitment strategy, authorized research personnel will search and contact potential research participants included within the member database of the Research Registry for study participation. This clinical trial will also be posted on the Research Registry website, as well as the public CAMH website. Once posted, interested participants can use the “Find a CAMH study” feature to explore clinical trials that they are interested in.

Exclusion criteria includes: 1) History of psychiatric illness (the Mini International Neuropsychiatric Interview (MINI) will be used to screen for psychiatric illness in the present or past), 2) Pre-diabetes or diabetes (fasting glucose ≥6.0 mmol/L, HbA1c>6% or use of anti-diabetic drug), 3) Evidence of impaired insulin sensitivity, assessed using the Homeostatic Model Assessment for Insulin Resistance (HOMA-IR) ≥2.5, 4) Family history of diabetes in a first degree relative (parent or sibling), 5) Use of weight reducing agents, 6) History of kidney or liver disease, 7) Moderate-to-severe substance use, 8) Irregular menstrual cycles (e.g., menstruation occurs less than 21 days or more than 35 days apart, or not having menstruated for three months (or 90 days), or conditions such as endometriosis or polycystic ovary syndrome (PCOS), or prior surgical interventions such as a hysterectomy or oophorectomy), 9) Use of hormonal birth control, 10) current use of progesterone, estrogen, testosterone, or fertility treatment, 11) Pregnant or breastfeeding, 12) Major medical or surgical event within the last 6 months, 13) any condition that interferes with safe acquisition of MRI data such as metal implants, pacemakers, cochlear implants, claustrophobia, etc., 14) any contraindications to the investigational products as listed in the product monographs including known hypersensitivity to the drug or the excipients of the product (note: enzymatic lactose intolerance is NOT exclusionary), 15) use of any of the prohibited medications listed in the product monograph of olanzapine (e.g., Levodopa and dopamine agonists and antihypertensive agents).

Additionally, these data will be compared with the subgroup of male participants from our previous study with the exact same inclusion criteria and imaging acquisition parameters to examine sex differences between males and females (REB #075/2017).

Prior to being asked to come in for a study screening visit, the prospective participant will be asked to complete a telephone pre-screen questionnaire, collecting minimal PHI, with a trained CAMH research personnel to assess their eligibility for the study.

The participant will receive compensation upon completion of each study visit (payment is made at each visit and cannot be paid in advance). The compensation schedule is below:

| **Visit** | **Amount ($ CAD)** |
| --- | --- |
| Screening Visit | $30 |
| MRI Visit 1 | $150 |
| MRI Visit 2 | $150 |
| MRI Visit 3 | $150 |
| MRI Visit 4 | $150 |
| End of study gratuity | $50 |
| **Total** | $680 |

TTC transit tickets/tokens, OR parking fees (if they drive) will be compensated at all visits. Participants will be asked to provide research staff with parking receipts.

A meal will be provided at the end of visits 1-4.

## 4.3 Equity, Diversity and Inclusion Considerations

The sample size of this study does not allow for gender considerations (e.g., gender identity and expression of the individual, institutional gender inequity experienced by the individual) to be accounted for fully. However, when appropriate, we will stratify randomization by gender identity and perform a sensitivity analysis by removing non-cisgender female participants.

Effects and trends of sex, gender, or race/ethnicity will be explored in larger future studies if data from this study supports the hypothesis and will be used as covariates in the analysis.

## 4.4 Eligibility Criteria

### 4.4.1 Inclusion Criteria

The female participant must meet all of the inclusion criteria to eligible for this clinical trial:

Inclusion criteria:

1. Must sign and date the informed consent form;
2. Stated willingness to comply with all study procedures;
3. Age: 18-35
4. Body Mass Index (BMI) between 18.5 and 24.9 kg/m^2^
5. Normal menstrual cycle (defined as cycle length ranging from 21 to 35 days over the past 6 months)

### 4.4.2 Exclusion Criteria

Female participants that meet any of the following criteria will be excluded from participation in this clinical trial:

Exclusion criteria for all participants:

1. History of psychiatric illness (screened using the Mini International Neuropsychiatric Interview (MINI))
2. Pre-diabetes or diabetes (fasting glucose ≥6.0 mmol/L, HbA1c>6% or use of anti-diabetic drug),
3. Evidence of impaired insulin sensitivity, assessed using the Homeostatic Model Assessment for Insulin Resistance (HOMA-IR) ≥2.5
   a. The following formula will be used to calculate HOMA-IR from fasting glucose (in mmol/L) and fasting insulin (in pmol/L): ((Insulin/6)*Glucose]/22.5) [60].
4. Family history of diabetes in a first degree relative (parent or sibling)
5. Use of weight reducing agents
6. History of kidney or liver disease
7. Moderate-to-severe substance use
8. Irregular menstrual cycles (e.g., menstruation occurs less than 21 days or more than 35 days apart, or not having menstruated for three months (or 90 days), or conditions such as endometriosis or polycystic ovary syndrome (PCOS) or prior surgical interventions such as a hysterectomy or oophorectomy)
9. Current use of hormonal birth control (e.g., pill, patch, hormonal intrauterine device [IUD], ring). Participants must have had at least 2 regular menstrual cycles following the discontinuation of hormonal birth control [1]
10. Current use of progesterone, estrogen, testosterone, or fertility treatment.
11. Pregnant, gave birth in the last year, or breastfeeding. Participants must have at least 3 regular menstrual cycles post-breastfeeding before beginning the study.
12. Major medical or surgical event within the last 6 months
13. Any condition that interferes with safe acquisition of MRI data such as metal implants, pacemakers, cochlear implants, claustrophobia, etc.
14. Any contraindications to the investigational products as listed in the product monographs including known hypersensitivity to the drug or the excipients of the product (note: enzymatic lactose intolerance is NOT exclusionary),
15. Use of any of the prohibited medications listed in the product monograph of olanzapine (e.g., Levodopa and dopamine agonists and antihypertensive agents).

## 4.5 Lifestyle Considerations

During this clinical trial, participants are asked to:

- Fast for 10 hours prior to any fasting bloodwork collections.
- Refrain from recreational drug use at least one week prior to any study visit.
- Participants will be asked to abstain from consuming alcohol for 24 hours prior to Day 0 and 24 hours after Day 2. Participants will be informed that olanzapine can exacerbate the side effects of alcohol consumption and that it is recommended that no alcohol be consumed while taking these medications.
- Agree to use an acceptable form of non-hormonal birth control (e.g. a barrier method of a diaphragm with spermicide and/or Latex condom, or copper intrauterine device) or a partner has had a vasectomy at least 3 months prior to study start or agreeing to abstain from sex for the duration of the trial.

## 4.6 Screen Failures

During the screening visit (following consent procedures), if the participant does not meet all inclusion criteria, or meets any of the exclusion criteria, the visit will be terminated. The participant will be informed that they, “Unfortunately, are not currently eligible for this study.” The outcomes off all screening visits will be documented in a “Screening Log” containing participants’ screening ID, date of consent, date of screening, and if they are eligible. If participants are ineligible for the study, the reason will also be indicated in the log.

## 4.7 Participant Withdrawal Criteria

### 4.7.1 When and How to Withdraw Participants

Participants are free to withdraw from participation in the clinical trial at any time.

An investigator may discontinue or withdraw a participant from the clinical trial for the following reasons:

- Pregnancy;
- Significant study intervention non-compliance;
- If any adverse event (AE), laboratory abnormality, or other medical condition or situation occurs such that continued participation in the clinical trial would not be in the best interest of the participant;
- Disease progression which requires discontinuation of the study intervention;
- If the participant meets an exclusion criterion (either newly developed or not previously recognized) that precludes further study participation.

The reason for participant discontinuation or withdrawal from the study will be recorded within the participant’s research record, and/or legal health record.

### 4.7.2 Follow-up for Withdrawn Participants

If a participant withdraws consent, information that was recorded prior to withdrawal, including the results of tests done on samples, will be kept by the researchers (it will not be destroyed), but no more information will be collected. Records of participation in this study will not be destroyed (for example, this signed consent form, and reports of study procedures that have been done). It will not be possible to withdraw results once they have been published or otherwise shared with the research community.

Once participants withdraw from the clinical trial, no further research procedures or evaluations will be performed, or additional research-specific data collected on the participant. Reasonable effort will be made to obtain permission to document the reason for withdrawal.

###### 4.7.3 Early Termination Visit

If a participant withdraws from the trial, the following procedures will be performed during this visit (if the participant consents to this visit):

- Assessment of new and ongoing AEs;
- Assessment of any complications following the study intervention;
- Documentation of all concomitant medications; and
- Performance of anthropometric measures and clinical scales.

The QI will ensure the participant is appropriately transitioned/followed for any additional care as required.

###### 4.7.4 Participants who are Lost to Follow-up

A participant will be considered lost to follow-up if they fail to return for the INI/OLA MRI visit and is unable to be contacted by the research team.

The following actions will be taken if a participant fails to attend a required study visit:

- The research team will attempt to contact the participant via the participant’s preferred method on a minimum of 3 separate days to (re)schedule the upcoming or missed visit. The participant will be counselled on the importance of maintaining the assigned visit schedule, and reconfirm whether the participant wishes to and/or should continue in the clinical trial.
- Before a participant is deemed lost to follow-up, the research team will make every effort to regain contact with the participant (where possible, three telephone calls, or emails on different days/instances). These contact attempts will be documented in the study’s “participant contact” log.
- Should the participant continue to be unreachable, they will be considered to have withdrawn from the clinical trial with a primary reason of lost to follow-up.

# 5.0 STUDY INTERVENTION

## 5.1 Description

The oral investigational agent used in this study is olanzapine (Generic brand: Apo-Olanzapine), self-administered at night over the course of two days (5mg and 10 mg, respectively). The intranasal investigational product will be either Insulin Lispro (100 IU/mL Humalog; Eli Lilly Canada) or placebo (0.9% sodium chloride injection USP) administered via a metered nasal dispenser (Medisca, Canada). A single spray dispenses 0.1 mL (10 IU) of insulin. A total of 160 IU of insulin (=1.6 mL) will be administered in Day 2 of each study visit (80 IU = 0.8 mL delivered per nostril). Similarly, 1.6 mL of the saline spray will be administered at each visit. The spray will be self-administered approximately 30 minutes prior to the start of the MRI scan at each visit.

## 5.2 Treatment Regimen

Small acute doses of OLA were chosen for reasons of practicality and safety, although this is not representative of how the drug is used clinically (i.e., daily administration, often at higher doses). Nonetheless, previous research has shown that a single 10 mg dose of OLA in healthy controls may invoke early changes in some parameters of glucose and lipid metabolism, justifying the chosen dose in this study [2].

Intranasal administration of insulin allows for it to bypass peripheral circulation and be delivered directly to the brain via the inulin receptors of the olfactory bulb.^58^ Therefore, this technique can separate peripheral versus central insulin action to elucidate the study aims. The use of 160 IU (=1.6 mL) has been previously used in adult studies, with the strongest dose-dependent response.^59^ The MRI protocol is completed within the 2-hour time limit of central insulin effects.^59^

## 5.3 Method for Assigning Participants to Treatment Groups

The study staff will confirm eligibility and assign a participant trial number. There is no randomization involved for the investigational products as all participants will receive oral placebo at visit 1 and oral olanzapine at visit 2 of each cycle phase. Study staff will be unblinded to ensure the proper scheduling of visits during each phase. Intranasal saline and intranasal insulin will be administered at both MRI visits.

## 5.4 Administration of Study Intervention

Participants will be instructed to take the oral OLA (or placebo) medication at night, at their home, on both day 0 and day 1. The medication should be taken at bedtime with water. Study staff will send a reminder to participants on Day 0 and Day 1 to take the medications and confirm once they have taken it and at what time. Participants will be asked to return the blister pack on Day 2 to be used for compliance. Serum OLA levels will also be measured on Visit 2 - Day 2 prior to MRI assessments to confirm compliance and to use as a covariate in analyses.

Insulin (100 IU/mL Humalog; Eli Lilly Canada), saline placebo (0.9% sodium chloride injection USP) solutions, and spray bottles will be supplied by the pharmacy. Research staff will transfer the solutions into metered nasal spray dispensers (15mL bottle, Medisca) on the day of the MRI scans. Participants will be instructed on how to properly administer the spray by study staff on the day of the MRI scan. The intranasal spray administration SOP (Procedure No. MA003) (adapted from Procedure No. SMA002 from study REB#075/2017) will be used to train staff on how to properly instruct participants.

- Prime the nasal spray bottle by spraying into the air a few times until mist/spray appears.
- Ask the subject to clear nose by blowing nose gently (provide participant with tissue).
- Ask participant to insert spray bottle into the nostril with their head tilted slightly back. Ensure that the bottle is upright.
- Ask participant to press a single spray from the metered dispenser into the nostril while they inhale. Repeat for second nostril.
- Set a timer for 60 seconds.
- At the end of one minute, similarly administer another spray into each nostril while participant inhales.
- Repeat this process another 6 times (for a total of 8 administrations into each nostril). Keep track of the time and number of administrations on a checklist.
- At each visit, a total of 160 IU (=1.6 mL) of placebo and insulin will be administered before MRI Scan #1 and Scan #2, respectively.
- Upon completion of the study visit, a trained staff member will return both the intranasal insulin or saline placebo vial, and the corresponding metered spray bottle to the CAMH pharmacy for disposal.

## 5.5 Participant Compliance Monitoring

Participants will be asked to return the blister pack on Day 2 to be used for compliance. Serum OLA levels will also be measured on Day 2 prior to MRI assessments to confirm compliance and use as a covariate in analyses.

Intranasal spray administration completed by participants will be monitored by research staff. Each administration, per nostril, will be noted in the study report forms. Any non-compliance will be noted. If the nasal spray is administered incorrectly, the participant will be re-trained by research staff.

## 5.6 Concomitant Therapy

Concomitant medical therapy will be collected at screening and used to assess participants’ eligibility for the study. The use of anti-diabetes medication and weight-reducing agents is not permitted during the clinical trial as it serves as a confounding factor for the primary analysis of the study. Concomitant medication/therapies will be verified with research participants at each visit.

## 5.7 Packaging

Oral OLA and PL will be dispensed by the pharmacy in identical looking capsules packaged in blister packs. INI or INP will be transferred from their respective vials into an unlabeled metered spray bottle that is identical for both conditions. Study staff will not be involved in any pharmacy related processes.

Insulin (100 IU/mL Humalog; Eli Lilly Canada) and saline placebo (0.9% sodium chloride injection USP) will be dispensed by the pharmacy in vials created by their manufacturer. The contents of the vials will be transferred to metered nasal spray dispenser (15mL bottle, Medisca), with no indication of the intervention on the bottle, by research staff on the day of the MRI visit. Research staff will mark the spray bottles with “Scan 1” or “Scan 2” to ensure participant blinding to the nasal spray condition and to be able to distinguish the contents of the identical bottles.

## 5.8 Blinding of Study Intervention

The study protocol follows a single-blind study design. Participants will be blinded to both the oral pill and the nasal spray condition. They will receive spray bottles with no indication of whether they are receiving insulin or placebo. A designated study staff will be responsible for transferring the insulin or saline into the nasal spray bottle. The medications will be dispensed in identical looking capsules (size and color).

## 5.9 Receiving, Storage, Dispensing and Return

### 5.9.1 Receipt of Study Intervention Supplies

Upon receipt of the study intervention supplies, an inventory will be performed, and a receipt log filled out and signed by the person accepting the shipment. Designated pharmacy staff must count and verify that the shipment contains all the items noted in the shipment inventory. Any damaged or unusable study intervention in a given shipment (active drug or comparator) will be documented in the clinical trial files. All IP will be maintained by the pharmacy. Research staff will return all used IP insulin (100 IU/mL Humalog; Eli Lilly Canada), saline placebo (0.9% sodium chloride injection USP) to the pharmacy.

### 5.9.2 Storage

The April 12, 2021 revised version of the Eli Lilly Canada Inc. Humalog product monograph states that: “Prior to first use, HUMALOG preparations must be stored in a refrigerator between 2° and 8°C. They should not be frozen or exposed to excessive heat or sunlight. Cartridges, vials, and prefilled pens that are in current use, should be stored at room temperature (below 30°C and away from direct heat and light) and discarded after 28 days. Do not use after expiry date on label.” The pharmacy will store the Humalog vials in refrigerated conditions prior to the MRI visit. After the Humalog prescription is filled, the vial will be stored at room temperature for less than 28 days before the MRI study visit.

Saline placebo (0.9% sodium chloride injection USP) will be stored at room temperature (25°C), away from heat and protected from freezing, by both the pharmacy and research team.

### 5.9.3 Dispensing of Study Intervention

A log of filled Olanzapine, Humalog and 0.9% sodium chloride prescriptions will be kept by research staff and pharmacy.

### 5.9.4 Return or Destruction of Study Intervention

At the completion of the clinical trial, there will be a final reconciliation of the study intervention shipped, consumed and remaining. This reconciliation will be logged on an accountability form, and signed and dated by delegated research and/or pharmacy staff. Any discrepancies noted will be investigated, resolved, and documented prior to return or destruction of unused study intervention. Intervention destroyed on site will be documented in the clinical trial’s files.

# 6.0 RESEARCH PROCEDURES

## 6.1 Research Visits

- Pre-Screening
  - Telephone screening questionnaire
- Screening Visit
  - Informed Consent
  - Review of medical history and demographics
  - Fasting bloodwork: these include fasting glucose and fasting insulin (to calculate HOMA-IR, a validated measure of peripheral insulin resistance), c-peptide, lipid profile, HbA1c, complete blood count (CBC), prolactin, vitamin B12, electrolytes, thyroid status, liver and kidney function tests.
  - Anthropometric measures: height, weight, blood pressure, waist circumference
  - Clinical questionnaires: the Mini-International Neuropsychiatric Interview (MINI) will be used to screen out the presence of psychiatric disorders.
  - Participants will be asked to report on their menstrual cycle and document their last 3 menstruation dates in order to determine average cycle duration and menstrual phases to schedule study visits. Progesterone, estrogen, LH and FSH levels will be measured as part of the bloodwork panel and will be used to confirm current menstrual cycle phase (follicular phase is defined as high estrogen levels, while the luteal phase is defined by high progesterone levels).
    - Once participants are enrolled in the study, they will be randomized to either the “follicular arm” or the “luteal arm”. This means that all study visits will take place in that specified menstrual phase. Participants in the follicular arm will be scanned between days 4-10 of their cycle, while participants in the luteal arm will be scanned between cycle days 16-22.
- Study Visit 1-4
- One week prior to the study visit, study staff will call the participant to ensure there have been no changes in their health since the last visit and that their menstrual cycle is still regular. They will be asked to confirm the date of their last menstrual cycle to ensure the study visit will be taking place in the proper phase.
- Participants will also be asked to refrain from any recreational drug use the week leading up to the MRI scan.
- *Day 0:*
  - Pregnancy test
  - Participant will take 5 mg of oral OLA or placebo on the nights of Day 0 or Day 1 before going to bed.
- *Day 1:*
  - Receive a standardized mixed meal (vegan and vegetarian options available). This can be eaten at the participants regular dinner time or until 9:30pm. This meal should be the participant’s last meal for the day, after which they are asked to remain fasted until the study visit is done the next day.
  - Complete the International Physical Activity Questionnaires (IPAQ)
  - Participants will take 10 mg of oral OLA (or PL) at night before going to bed and confirm with study staff once medications have been taken.
- *Day 2:*
  - Fasting bloodwork: blood glucose, insulin, c-peptide, estrogen, progesterone, LH, FSH and OLA levels.
  - Bloodwork (glucose, insulin and c-peptide) will be repeated after the scan
  - Intranasal placebo administration followed by MRI Scan #1 (~30 minutes)
  - Intranasal insulin administration followed by MRI Scan #2 (~30 minutes)
  - Visual Analogue Scale (VAS) and Food Craving Questionnaires (FCQ); administered before intranasal placebo is administered and again after intranasal insulin is administered.
  - Cognitive testing: Brief Visuospatial Memory Test Revised of the Kaplan Function System, Digit Symbol Substitution Test
  - Scales and questionnaires: 3-day food diary (returned by participant), Food Craving Scales, Profile of Mood State (POMS) scale, Subjective Wellbeing Under Neuroleptics (SWN), Barnes Akathesia Rating Scale (BAS), Stanford Sleepiness Scale (SSS).
  - Anthropometric measures (height, weight, waist circumference, blood pressure)

## 6.2 Schedule of Events (Figure 1)

| **Assessments** | **Screen** | **1** | **2** | **3** | **4** |
| --- | --- | --- | --- | --- | --- |
| Informed Consent/Assent | X |  |  |  |  |
| Demographics form | X |  |  |  |  |
| Eligibility/Termination Checklist | X |  |  |  |  |
| MINI | X |  |  |  |  |
| Edinburgh Handedness Questionnaire | X |  |  |  |  |
| Anthropometric Measures (weight, height, waist circumference) | X |  |  |  |  |
| Fasting Bloodwork | X |  |  |  |  |
| Olanzapine administration (on day 0, and 1 of each visit) |  | X | X | X | X |
| MRI scanning and cognitive testing (on day 2 of each visit) |  | X | X | X | X |
| Stanford Sleepiness scale (Day 2) |  | X | X | X | X |
| Post procedure Bloodwork (Day 2) |  | X | X | X | X |
| 3-Day Dietary History (to be returned on Day 2) |  | X | X | X | X |
| IPAQ (Day 1) |  | X | X | X | X |
| FCQ and VAS (Day 2) |  | X | X | X | X |
| BAS (Day 2) |  | X | X | X | X |
| POMS (Day 2) |  | X | X | X | X |
| SWN (Day 2) |  | X | X | X | X |
| Urine Sample (drug screen* + HCG)  * Urine drug screen done at visits 1-4 per PI discretion if positive drug test at screening or positive self-report. | X | X | X | X | X |

**Figure 3.** Each visit will have two MRI scans, the first following INP administration, and the second following INI administration. Study visits will take place 2-6 weeks apart to ensure specific menstrual phase across the four visits. Participants will undergo two visits during the follicular phase of the menstrual cycle (they will be scanned between day 4-10 of their menstrual cycle), and another two visits during the luteal phase of their menstrual cycle (between day 16-22, or within 5 days of next expected menses depending on individual cycle duration). There will be a lenience of +/- 1 hour for study assessment start time on day 2.

# 7.0 STATISTICAL PLAN

## 7.1 Sample Size Determination

This study examines whether brain insulin action differs in females according to their menstrual cycle phase, and how a high metabolic liability agent such as OLA might interrupt these differential insulin effects. Previous studies investigating the role of INI on MRI measures ^34,^ ^35,^ ^37, 58^ including our previous study show medium to large effect sizes (Cohen’s d: 0.5 to 1.4, f: 0.4 to 0.6). N=15 would be sufficient to detect a medium to large effect with 80% power and α=0.05.

## 7.2 Statistical Methods

MRI data analysis: Resting state fMRI: The Data Processing Assistant for Resting-State fMRI (DPARSF) (<http://www.restfmri.net>) will be used to analyze the resting state fMRI data to obtain fractional amplitude of low-frequency fluctuation (fALFF) for the regions of interest (DLPFC, striatum, and hippocampus) and CONN (<https://web.conn-toolbox.org/home>) Toolbox will be used for analysis of functional connectivity.

Statistical analysis: Mixed Effect Models will be used to investigate the impact of menstrual phase on the effect of INI in females, and the additive effect of OLA on this response. The three treatment conditions, group (i.e., follicular phase vs. luteal phase), and time, will be entered as predictors and participants will be used as random factors in a random intercept model. Age, BMI, and sedation levels (due to OLA intake) will be used as covariates in the analysis. Additionally, these data will be compared with the subgroup of male participants from our previous study with the exact same inclusion criteria and imaging acquisition parameters to examine sex effects ^41^.

Significance is defined as p-value ≤0.05. No interim analysis will be conducted for the termination of the trial. All participants enrolled in the study and that complete all three research visits will be analyzed. However, participants who have missing data from the research visits will not be included for that part of the analysis. Instead, other collected data will be used for primary/secondary analysis, where applicable.

# 8.0 SAFETY AND ADVERSE EVENTS

## 8.1 Definitions

Adverse Event

An **adverse event** (AE) is any untoward medical occurrence in a research participant administered an investigational product and which does not necessarily have a causal relationship with this treatment. An adverse event (AE) can therefore be any unfavorable and unintended sign (including an abnormal laboratory finding), symptom, or disease temporally associated with the use of a medicinal (investigational) product, whether or not related to the investigational product.

**Serious Adverse Event**

A **serious adverse event** (SAE) is any AE that is:

- Fatal;
- Life-threatening;
- Requires or prolongs hospital stay;
- Results in persistent or significant disability or incapacity;
- A congenital anomaly or birth defect; or
- An important medical event (events that may not be life threatening but are of major clinical significance, such as a drug overdose or seizure that did not result in in-patient hospitalization).

Adverse Event Collection Period

The period during which adverse events must be collected is normally defined as the period from the initiation of any research procedures to the end of the study treatment follow-up. For this clinical trial, the study treatment follow-up is defined as 30 days following the last administration of the study intervention.

AEs occurring as of the first administered dose of the study intervention and 30 days after the last administered dose will be collected. AEs recorded during this period will be followed through to resolution, or until the event is assessed as chronic or stable.

Preexisting Condition

Medical history and diagnoses will be collected and recorded during the screening visit, and any clinically significant abnormality will be recorded as a preexisting condition. If the frequency, intensity, or the character of the condition worsens during the study period, this will be recorded as an adverse event. Any new clinically significant findings/abnormalities that meet the definition of an adverse event will also be recorded and documented as an adverse event.

Post-study Adverse Event

At the last scheduled visit, the PI and/or QI should instruct each participant to report any subsequent event(s) that the participant believes might reasonably be related to participation in this clinical trial. The PI and/or QI should notify the study sponsor of any death or adverse event occurring at any time after a participant has discontinued or terminated participation that may reasonably be related to this clinical trial. The sponsor should also be notified if the PI and/or QI should become aware of the development of cancer or of a congenital anomaly in a subsequently conceived offspring of a participant that was involved in this clinical trial.

Abnormal Laboratory Values

A clinical laboratory abnormality will be documented as an adverse event if any one of the following conditions are met:

- The laboratory abnormality is not otherwise refuted by a repeat test to confirm the abnormality;
- The abnormality suggests a disease and/or organ toxicity;
- The abnormality is of a degree that requires active management (e.g. change of dose, discontinuation of the study intervention, more frequent follow-up assessments, further diagnostic investigation, etc.); or
- Any laboratory abnormalities assessed as being clinically significant by a study physician or qualified individual.

## 8.2 Recording of Adverse Events active language

*All adverse events occurring during the study period will be recorded. At each contact* with the research participant, the research team will seek information on adverse events by specific questioning and, as appropriate, by examination. Information on all adverse events will be recorded immediately in the participant’s research record and/or legal health record, and transcribed into the adverse event log. All adverse events will be assessed by a qualified individual for relatedness, expectedness, seriousness, and severity in relation to the study intervention.

All clearly related signs, symptoms, and abnormal diagnostic procedure results will be recorded in the research record and/or legal health record and assessed by a qualified individual in a timely manner allowing sufficient time to meet required reporting timelines for SAEs and SUADRs if needed.

## 8.3 Reporting of Serious Adverse Events

### 8.3.1 Investigator Reporting: Notifying the Sponsor

Reporting SAEs to the sponsor will occur within 24 hours of becoming aware of the event. SOP # **HSR 207 - Serious Adverse Event (SAE) Reporting** will be followed for the reporting of SAEs.

### 8.3.2 Investigator Reporting: Notifying the REB

The process for notification to the REB for applicable SAEs must be completed as per REB reporting requirements. SAEs and unanticipated events must be recorded and reported to the REB in accordance with the REB’s reporting requirements and timelines. Copies of each report and documentation of REB notification and REB receipt/acknowledgement must be kept in the Investigator Study Binder.

### 8.3.3 Sponsor Reporting of SUADRs: Notifying Health Canada

***SOP # HSR 225 – Serious Unexpected Adverse Drug Reaction (SUADR)*** *will be followed for the reporting of SUADRs to Health Canada as per Division 5.*

*The* [*Council for International Organizations of Medical Sciences (CIOMS)*](https://cioms.ch/cioms-i-form/) *form will be used to submit a SUADR report to Health Canada.*

### 8.3.4 Sponsor Reporting of SUADRs: Notifying Sites

N/A.

## 8.4 Reporting of Device Deficiencies

N/A.

*For medical device clinical trials only: define the process and timelines for reporting device deficiencies to the sponsor and manufacturer.*

## 8.5 Safety Management Plan

Reasons for withdrawing individual participants from the study may include one or more of the following:

a) Major protocol violation
b) Participant lost to follow-up
c) Withdrawal of consent

Concomitant medications and side-effects are assessed at each study visit. Changes to medications and health conditions will be reviewed by the PI. If the changes lead to the participant falling under the exclusion criteria for the study, the participant will be withdrawn from the study.

Any participant may be discontinued from the study at the discretion of the investigators if this is deemed to be in the best interest of the participant. The decision may be made either to protect the participant’s health and safety, or because it is part of the research plan that people who develop certain conditions may not continue to participate.

## 8.6 Unblinding Procedures

Participants will not be unblinded to the oral medication or spray contents, as the study follows a crossover design, such that they will receive all conditions while participating in the study. In the case of SAEs, participants will be unblinded to the condition.

## 8.7 Data and Safety Monitoring Board

N/A

# 9.0 CLINICAL TRIAL DISCONTINUATION AND CLOSURE

## 9.1 Clinical Trial Discontinuation

This clinical trial may be temporarily suspended or prematurely terminated if there is sufficient reasonable cause (i.e. closure based on PI decision, sponsor/funder decision, REB or other oversight bodies’ decision; review of serious, unexpected and related AEs; noncompliance; futility). Notification, which includes the reason for study suspension or termination, will be provided by the suspending or terminating party to research participants, the PI, funding agency, CAMH, and regulatory authorities. If the clinical trial is prematurely terminated or suspended, the PI will promptly inform research participants, the REB, and the sponsor, and will provide the reason(s) for the termination or suspension. All communication with participants for this purpose will go through REB review and approval. Research participants will then be contacted, as applicable, and be informed of changes to the study visit schedule.

# 10.0 DATA HANDLING AND RECORD KEEPING

## 10.1 Source Documents & Case Report Forms

Data will be collected by a trained study personnel on paper copies of the Case/Data Report Forms. Data from the CRFs will then be entered into REDCap electronic case report forms, where they will be validated by a second trained study personnel. This system is maintained on central CAMH servers, with data backed up daily, and is supported by the Research Informatics department.

## 10.2 Protocol Deviations

No deviations from or changes to the protocol will be implemented without prior agreement from the sponsor as required, and approval from the REB, unless to eliminate an immediate hazard to a participant.

## 10.3 Record Retention

As per the Health Canada retention policy, all study- related personal health information will be retained for the required 15 years. Confidentiality will be respected and no information that discloses the participant’s identity will be released or published without consent, unless required by law.

## 10.4 Clinical Trial Registration

In accordance with TCPS 2, a description of the clinical trial will be registered in a publicly accessible database (e.g. www.clinicaltrials.gov) before the start of recruitment activities, and the content will be updated throughout the duration of the clinical trial. All results, including negative results must be entered at the completion of the clinical trial.

# 11.0 STUDY MONITORING, AUDITING, AND INSPECTING

## 11.1 Study Monitoring Plan

Study monitoring for this study will be conducted by the Quality Assurance office at CAMH on pre-determined intervals (i.e. after a specified number of participants complete the study or yearly, whichever comes first) this will be outlined within the study’s Monitoring Plan which will be reviewed and signed by the PI and other relevant parties. The basic protection against risk in this study will be provided by the PI. The PI or documented designate will have primary responsibility for monitoring of participants during the entire time they participate in the study. The PI will meet regularly with study personnel to review accrued data, data confidentiality, and adherence to protocol design, recruitment, and participant complaints. During meetings the Study PI will also review the enrollment data, the accrual and integrity of clinical data, and any adverse event associated with the various components of the study.

Site monitoring will be conducted to ensure that the rights and well-being of research participants are protected, the reported trial data are accurate, complete, and verifiable, and the conduct of the clinical trial is in compliance with the currently approved protocol/amendment(s), ICH GCP, and applicable regulatory requirement(s).

## 11.2 Auditing and Inspecting

The PI and site will permit study-related audits, and inspections by the REB, CAMH, sponsor, and applicable granting agencies or regulatory bodies, including access to all study-related documents (e.g. source documents, regulatory documents, data collection instruments, study data, etc.). The PI will ensure the capability for audits/inspections of applicable study-related facilities (e.g. research pharmacy, clinical laboratory, imaging facility, etc.).

# 12.0 ETHICAL CONSIDERATIONS

Clinical trial materials (e.g., protocol, ICF, recruitment materials, written information provided to participants, etc.) will be submitted to the research ethics board (REB) for review and approval in accordance with REB requirements. Approval will be obtained prior to initiating any clinical trial-specific tasks, and maintained throughout the course of the clinical trial in accordance with REB requirements. Any amendments will require review and approval by the REB before the changes are implemented in the clinical trial, unless to eliminate an immediate hazard to the participant. The REB will be notified of any unanticipated issue or event that may increase the level of risk to participants or that has other ethical implications that may affect participants’ welfare.

## 12.1 Research Ethics Board (REB) Approval

Research Ethics Board (REB) approval will be obtained prior to beginning any research-specific procedures. Following initial ethics approval, ongoing ethical approval will be maintained and the clinical trial will undergo REB review at least annually, in accordance with regulatory and REB requirements. The clinical trial will be conducted in accordance with the REB-approved study documents and the determinations (including any limitations) of the REB, and in compliance with REB requirements.

Whenever new information becomes available that may be relevant to participant consent, a consent form and/or consent for addendum will be presented to the REB for review and approval prior to its use. Any revised written information will receive REB approval prior to use.

## 12.2 Informed Consent Process & Documentation

Informed consent is a process that is initiated prior to the individual agreeing to take part in the clinical trial and continues throughout their participation.

Informed consent will be obtained from each participant prior to their participation in the clinical trial. Informed consent will be obtained by appropriately trained and qualified CAMH research personnel who do not have an existing clinical relationship with the participant. The PI will not obtain participant consent. Each participant will be provided with a current copy of the REB approved ICF prior to the consent discussion. Research personnel will explain the clinical trial to the participant and answer any questions that may arise. This discussion will include an explanation of the clinical trial purpose, procedures, potential risks and benefits, confidentiality considerations and participant rights (e.g., participants will not be penalized or lose any benefits regardless of what they decide, and they have the right to withdraw from the clinical trial at any time). The MacCAT-CR will be administered to ensure participants can provide consent**.**

Participants may take as much time as they need to make their decision, and may consult with others (e.g., family members, other health care providers, etc.) if they like. Following the consent discussion, and once the participant has decided to take part, the participant and the person conducting the consent discussion will personally sign and date the ICF. Each participant/ will be provided with a complete (fully signed) copy of the ICF. The original ICF(s) and the informed consent process will be documented in the source documents.

# 13.0 PRIVACY AND CONFIDENTIALITY

All clinical trial-related documents and data will be held in strict confidence and stored at CAMH or on CAMH servers, and will follow CAMH policies and procedures to ensure participant privacy and confidentiality. All information that identifies the participant will be kept confidential and stored and locked in a secure place that only authorized personnel will have access to. In addition, electronic files will be stored on a secure hospital or institutional network and will be password protected. The investigators will dispose of the paper and computer-based records after the research obligations for the study have been met. As per the Health Canada retention policy, all study- related personal health information will be retained for the required 10 years. Confidentiality will be respected and no information that discloses the participant’s identity will be released or published without consent, unless required by law.

There are three exceptions to our confidentiality policy. In any of the following situations, we are obligated by law to contact authorities: 1) if there is a serious possibility that the person may harm themself or others, 2) if they have been involved in any form of child abuse or neglect, 3) if they have been the victim of abuse by a healthcare worker.

As part of the Research Services Quality Assurance Program, this study may be monitored and/or audited by a member of the Quality Assurance Team. The participant’s research records may be reviewed during which confidentiality will be maintained as per CAMH policies and extent permitted by law.

As part of continuing review of the research, participant study records may be assessed on behalf of the Research Ethics Board. A person from the research ethics team may contact the participant (if their contact information is available) to ask questions about the research study and consent to participate. The person assessing the file or contacting the participant must maintain your confidentiality to the extent permitted by law.

All research activities will be conducted in as private a setting as possible. The study monitor, other authorized representatives of the sponsor, representatives of the REB, regulatory agencies may inspect all documents and records required to be maintained by the PI, including but not limited to, medical records and pharmacy records for the participants in this clinical trial.

The information provided will not affect the usual care that the participant receives. To protect participant identity, the research records will be indicated by a code number rather than by name, and the information linking these case numbers with participant identity will be kept separate from the research records. The information linking this code number to participant identity will be kept in a separate, secure location and will only be accessible to the study team. The study team may also access participant health records for research purposes; their medical records will be kept confidential.

Following the completion of the study, the researchers intend to publish the results in scientific journals. Participants will not be identified in any of these reports. A report of the results of this project will be given to participants if requested during interviews.

Please note, the security of information sent by e-mail cannot be guaranteed. Please do not communicate personal sensitive information by e-mail. E-mail is not monitored outside of work hours. The participant will be advised not to use e-mail to communicate emergency or urgent health matters – and to please contact their clinician or, in case of a medical emergency, call 911.

Confidentiality will be broken if the participant is judged to be a danger to themself or another; if they disclose abuse from a healthcare worker, or if court ordered. Other laboratories (other than CAMH) may be involved in analyzing the data, and if so, this will be confidential, and the participant’s name will not be given out.

# 14.0 CLINICAL TRIAL FINANCES

## 14.1 Funding Source

This study is funded by Womenmind 2023 Seed Funding Competition.

## 14.2 Conflict of Interest

There are no conflicts of interest of any investigator with this clinical trial.

# 15.0 PUBLICATION POLICY/DATA SHARING

The CAMH is solely responsible for maintaining information relevant to the patients’ participation.

The PI will hold primary responsibility for publication of the results of this clinical trial. Any use or sharing of information from this study will need to be authorized by the study PI.

## 15.1 Future Secondary Use of Data

De-identified data from this project may be used for future research by internal and/or external project collaborators with the authorization of the study PI.

# 16.0 REFERENCES

1.	Tandon, R., Nasrallah, H. A. & Keshavan, M. S. Schizophrenia, ‘Just the Facts’ 5. Treatment and prevention Past, present, and future. *Schizophrenia Research* vol. 122 Preprint at <https://doi.org/10.1016/j.schres.2010.05.025> (2010).

2.	Di Pietro, N. & Illes, J. Rising antipsychotic prescriptions for children and youth: Cross-sectoral solutions for a multimodal problem. *CMAJ. Canadian Medical Association Journal* vol. 186 Preprint at <https://doi.org/10.1503/cmaj.131604> (2014).

3.	Vancampfort, D. *et al.* Risk of metabolic syndrome and its components in people with schizophrenia and related psychotic disorders, bipolar disorder and major depressive disorder: A systematic review and meta-analysis. *World Psychiatry* vol. 14 Preprint at <https://doi.org/10.1002/wps.20252> (2015).

4.	Rajkumar, A. P. *et al.* Endogenous and antipsychotic-related risks for diabetes mellitus in young people with schizophrenia: A danish population-based cohort study. *American Journal of Psychiatry* **174**, (2017).

5.	Hennekens, C. H., Hennekens, A. R., Hollar, D. & Casey, D. E. Schizophrenia and increased risks of cardiovascular disease. *American Heart Journal* vol. 150 Preprint at <https://doi.org/10.1016/j.ahj.2005.02.007> (2005).

6.	Stogios, N. *et al.* Weight Gain and Metabolic Adverse Effects of Off-Label Second-Generation Antipsychotic Use in the Adult Population: A Systematic Review and Meta-Analysis. *Biol Psychiatry* **89**, (2021).

7.	Correll, C. U., Detraux, J., De Lepeleire, J. & De Hert, M. Effects of antipsychotics, antidepressants and mood stabilizers on risk for physical diseases in people with schizophrenia, depression and bipolar disorder. *World Psychiatry* **14**, (2015).

8.	Musil, R., Obermeier, M., Russ, P. & Hamerle, M. Weight gain and antipsychotics: A drug safety review. *Expert Opinion on Drug Safety* vol. 14 Preprint at <https://doi.org/10.1517/14740338.2015.974549> (2015).

9.	Laursen, T. M., Munk-Olsen, T. & Vestergaard, M. Life expectancy and cardiovascular mortality in persons with schizophrenia. *Current Opinion in Psychiatry* vol. 25 Preprint at <https://doi.org/10.1097/YCO.0b013e32835035ca> (2012).

10.	Correll, C. U. *et al.* Cardiometabolic risk in patients with first-episode schizophrenia spectrum disorders baseline results from the RAISE-ETP study. *JAMA Psychiatry* **71**, (2014).

11.	Preclinical and Clinical Sex Differences in Antipsychotic-Induced Metabolic Disturbances: A Narrative Review of Adiposity and Glucose Metabolism. *J Psychiatr Brain Sci* (2019) doi:10.20900/jpbs.20190013.

12.	Kraal, A. Z., Ward, K. M. & Ellingrod, V. L. Sex differences in antipsychotic related metabolic functioning in schizophrenia spectrum disorders. *Psychopharmacol Bull* **47**, (2017).

13.	McEvoy, J. P. *et al.* Prevalence of the metabolic syndrome in patients with schizophrenia: Baseline results from the Clinical Antipsychotic Trials of Intervention Effectiveness (CATIE) schizophrenia trial and comparison with national estimates from NHANES III. *Schizophr Res* **80**, (2005).

14.	Werner, F. M. & Coveñas, R. Safety of antipsychotic drugs: Focus on therapeutic and adverse effects. *Expert Opinion on Drug Safety* vol. 13 Preprint at <https://doi.org/10.1517/14740338.2014.935761> (2014).

15.	Bora, E., Akdede, B. B. & Alptekin, K. The relationship between cognitive impairment in schizophrenia and metabolic syndrome: A systematic review and meta-analysis. *Psychological Medicine* vol. 47 Preprint at <https://doi.org/10.1017/S0033291716003366> (2017).

16.	Rotstein, A., Shadmi, E., Roe, D., Gelkopf, M. & Levine, S. Z. Gender differences in quality of life and the course of schizophrenia: national study. *BJPsych Open* **8**, (2022).

17.	Grajales, D., Ferreira, V. & Valverde, Á. M. Second-generation antipsychotics and dysregulation of glucose metabolism: Beyond weight gain. *Cells* vol. 8 Preprint at <https://doi.org/10.3390/cells8111336> (2019).

18.	Sacher, J. *et al.* Effects of olanzapine and ziprasidone on glucose tolerance in healthy volunteers. *Neuropsychopharmacology* **33**, (2008).

19.	Teff, K. L. *et al.* Antipsychotic-induced insulin resistance and postprandial hormonal dysregulation independent of weight gain or psychiatric disease. *Diabetes* **62**, (2013).

20.	Chintoh, A. F. *et al.* Insulin resistance and secretion in vivo: Effects of different antipsychotics in an animal model. *Schizophr Res* **108**, (2009).

21.	Smith, G. C., Chaussade, C., Vickers, M., Jensen, J. & Shepherd, P. R. Atypical antipsychotic drugs induce derangements in glucose homeostasis by acutely increasing glucagon secretion and hepatic glucose output in the rat. *Diabetologia* **51**, (2008).

22.	Hahn, M. K. *et al.* Acute effects of single-dose olanzapine on metabolic, endocrine, and inflammatory markers in healthy controls. *J Clin Psychopharmacol* **33**, (2013).

23.	Albaugh, V. L., Singareddy, R., Mauger, D. & Lynch, C. J. A double blind, placebo-controlled, randomized crossover study of the acute metabolic effects of olanzapine in healthy volunteers. *PLoS One* **6**, (2011).

24.	Hahn, M. K. *et al.* Effects of intracerebroventricular (ICV) olanzapine on insulin sensitivity and secretion in vivo: An animal model. *European Neuropsychopharmacology* **24**, (2014).

25.	Kullmann, S. *et al.* Brain insulin resistance at the crossroads of metabolic and cognitive disorders in humans. *Physiol Rev* **96**, (2016).

26.	Agarwal, S. M. *et al.* Brain insulin action in schizophrenia: Something borrowed and something new. *Neuropharmacology* vol. 163 Preprint at <https://doi.org/10.1016/j.neuropharm.2019.05.010> (2020).

27.	Obici, S., Zhang, B. B., Karkanias, G. & Rossetti, L. Hypothalamic insulin signaling is required for inhibition of glucose production. *Nat Med* **8**, (2002).

28.	Filippi, B. M., Yang, C. S., Tang, C. & Lam, T. K. T. Insulin activates Erk1/2 signaling in the dorsal Vagal complex to inhibit glucose production. *Cell Metab* **16**, (2012).

29.	Dash, S., Xiao, C., Morgantini, C., Koulajian, K. & Lewis, G. F. Intranasal insulin suppresses endogenous glucose production in humans compared with placebo in the presence of similar venous insulin concentrations. *Diabetes* **64**, (2015).

30.	Kowalchuk, C. *et al.* In male rats, the ability of central insulin to suppress glucose production is impaired by olanzapine, whereas glucose uptake is left intact. *Journal of Psychiatry and Neuroscience* **42**, (2017).

31.	Benoit, S., Schwartz, M., Baskin, D., Woods, S. C. & Seeley, R. J. CNS melanocortin system involvement in the regulation of food intake. *Horm Behav* **37**, (2000).

32.	Hallschmid, M. *et al.* Intranasal insulin reduces body fat in men but not in women. *Diabetes* **53**, (2004).

33.	Clegg, D. J., Brown, L. M., Woods, S. C. & Benoit, S. C. Gonadal hormones determine sensitivity to central leptin and insulin. *Diabetes* **55**, (2006).

34.	Kullmann, S. *et al.* Intranasal insulin enhances brain functional connectivity mediating the relationship between adiposity and subjective feeling of hunger. *Sci Rep* **7**, (2017).

35.	Kullmann, S. *et al.* Dose-dependent effects of intranasal insulin on resting-state brain activity. *Journal of Clinical Endocrinology and Metabolism* **103**, (2018).

36.	Novak, V. *et al.* Enhancement of vasoreactivity andcognition by intranasal insulin in Type 2 diabetes. *Diabetes Care* **37**, (2014).

37.	Karczewska-Kupczewska, M. *et al.* The effect of insulin infusion on the metabolites in cerebral tissues assessed with proton magnetic resonance spectroscopy in young healthy subjects with high and low insulin sensitivity. *Diabetes Care* **36**, (2013).

38.	Caravaggio, F. *et al.* Reduced insulin sensitivity may be related to less striatal glutamate: An 1 H-MRS study in healthy non-obese humans. *European Neuropsychopharmacology* **28**, (2018).

39.	De La Fuente-Sandoval, C. *et al.* Glutamate levels in the associative striatum before and after 4 weeks of antipsychotic treatment in first-episode psychosis: A longitudinal proton magnetic resonance spectroscopy study. *JAMA Psychiatry* **70**, (2013).

40.	Fuente-Sandoval, C. D. La *et al.* Higher levels of glutamate in the associative-striatum of subjects with prodromal symptoms of schizophrenia and patients with first-episode psychosis. *Neuropsychopharmacology* **36**, (2011).

41.	Stogios, N. *et al.* Investigating the effects of antipsychotics on brain insulin action: Study protocol for a multi-modality magnetic resonance imaging (MRI) study in healthy controls. *PLoS One* **17**, (2022).

42.	Benedict, C. *et al.* Intranasal insulin improves memory in humans: Superiority of insulin aspart. *Neuropsychopharmacology* **32**, (2007).

43.	Hallschmid, M., Benedict, C., Schultes, B., Born, J. & Kern, W. Obese men respond to cognitive but not to catabolic brain insulin signaling. *Int J Obes* **32**, (2008).

44.	Heni, M. *et al.* Central insulin administration improves whole-body insulin sensitivity via hypothalamus and parasympathetic outputs in men. *Diabetes* **63**, (2014).

45.	Heni, M. *et al.* Insulin Action in the Hypothalamus Increases Second-Phase Insulin Secretion in Humans. *Neuroendocrinology* **110**, (2020).

46.	Kullmann, S. *et al.* Central Insulin Modulates Dopamine Signaling in the Human Striatum. *Journal of Clinical Endocrinology and Metabolism* **106**, (2021).

47.	Benedict, C., Kern, W., Schultes, B., Born, J. & Hallschmid, M. Differential sensitivity of men and women to anorexigenic and memory-improving effects of intranasal insulin. *Journal of Clinical Endocrinology and Metabolism* **93**, (2008).

48.	Hallschmid, M., Higgs, S., Thienel, M., Ott, V. & Lehnert, H. Postprandial administration of intranasal insulin intensifies satiety and reduces intake of palatable snacks in women. *Diabetes* **61**, (2012).

49.	BENKENDORFF, C. F. C. *et al.* 150-OR: Brain Insulin Sensitivity Is Modulated by Menstrual Cycle. *Diabetes* **69**, (2020).

50.	Yeung, E. H. *et al.* Longitudinal study of insulin resistance and sex hormones over the menstrual cycle: The biocycle study. *Journal of Clinical Endocrinology and Metabolism* **95**, (2010).

51.	Pulido, J. M. E. & Salazar, M. A. Changes in insulin sensitivity, secretion and glucose effectiveness during menstrual cycle. *Arch Med Res* **30**, (1999).

52.	Pletzer, B., Harris, T. A., Scheuringer, A. & Hidalgo-Lopez, E. The cycling brain: menstrual cycle related fluctuations in hippocampal and fronto-striatal activation and connectivity during cognitive tasks. *Neuropsychopharmacology* **44**, (2019).

53.	Schneider, E. *et al.* The effect of intranasal insulin on appetite and mood in women with and without obesity: an experimental medicine study. *Int J Obes* **46**, (2022).

54.	Kullmann, S. *et al.* Intranasal insulin modulates intrinsic reward and prefrontal circuitry of the human brain in lean women. *Neuroendocrinology* **97**, (2013).

55.	Benedict, R. H. B., Schretlen, D., Groninger, L., Dobraski, M. & et al. Revision of the Brief Visuospatial Memory Test: Studies of normal performance, reliability, and validity. *Psychol Assess* **8**, (1996).

56.	Hoddes, E., Zarcone, V., Smythe, H., Phillips, R. & Dement, W. C. Quantification of Sleepiness: A New Approach. *Psychophysiology* **10**, (1973).

57.	McLeod, D. R., Griffiths, R. R., Bigelow, G. E. & Yingling, J. An automated version of the digit symbol substitution test (DSST). *Behavior Research Methods & Instrumentation* **14**, (1982).

58.	Hallschmid, M. Intranasal insulin. *J Neuroendocrinol* **33**, e12934 (2021).

59.	Kullmann, S. *et al.* Dose-Dependent Effects of Intranasal Insulin on Resting-State Brain Activity. *J Clin Endocrinol Metab* **103**, 253–262 (2018).

60. Muniyappa R, Madan R, Varghese RT. Assessing Insulin Sensitivity and Resistance in Humans. [Updated 2021 Aug 9]. In: Feingold KR, Anawalt B, Blackman MR, et al., editors. Endotext [Internet]. South Dartmouth (MA): MDText.com, Inc.; 2000-. Available from: https://www.ncbi.nlm.nih.gov/books/NBK278954/

1.	Schmalenberger, K.M., H.A. Tauseef, J.C. Barone, S.A. Owens, L. Lieberman, M.N. Jarczok, S.S. Girdler, J. Kiesner, B. Ditzen, and T.A. Eisenlohr-Moul, *How to study the menstrual cycle: Practical tools and recommendations.* Psychoneuroendocrinology, 2021. **123**: p. 104895.

2.	Hahn, M.K., T.M. Wolever, T. Arenovich, C. Teo, A. Giacca, V. Powell, L. Clarke, P. Fletcher, T. Cohn, R.S. McIntyre, S. Gomes, A. Chintoh, and G.J. Remington, *Acute Effects of Single-Dose Olanzapine on Metabolic, Endocrine, and Inflammatory Markers in Healthy Controls.* J Clin Psychopharmacol, 2013.
